# Platinum‐Catalysed Selective Aerobic Oxidation of Methane to Formaldehyde in the Presence of Liquid Water

**DOI:** 10.1002/anie.202206841

**Published:** 2022-08-16

**Authors:** Sinqobile V. L. Mahlaba, Nasseela Hytoolakhan Lal Mahomed, Alisa Govender, Junfeng Guo, Gerard M. Leteba, Pierre L. Cilliers, Eric van Steen

**Affiliations:** ^1^ Catalysis Institute Department of Chemical Engineering University of Cape Town Private Bag X3 Rondebosch 7701 South Africa; ^2^ Group Technology Sasol South Africa (Pty) Ltd. P.O. Box 1 Sasolburg 1947 South Africa

**Keywords:** Formaldehyde, Methane, Methanediol, Oxidation, Platinum

## Abstract

The aerobic, selective oxidation of methane to C_1_‐oxygenates remains a challenge, due to the more facile, consecutive oxidation of formed products to CO_2_. Here, we report on the aerobic selective oxidation of methane under continuous flow conditions, over platinum‐based catalysts yielding formaldehyde with a high selectivity (reaching 90 % for Pt/TiO_2_ and 65 % over Pt/Al_2_O_3_) upon co‐feeding water. The presence of liquid water under reaction conditions increases the activity strongly attaining a methane conversion of 1–3 % over Pt/TiO_2_. Density‐functional theory (DFT) calculations show that the preferential formation of formaldehyde is linked to the stability of the di‐σ‐hydroxy‐methoxy species on platinum, the preferred carbon‐containing species on Pt(111) at a high chemical potential of water. Our findings provide novel insights into the reaction pathway for the Pt‐catalysed, aerobic selective oxidation of CH_4_.

## Graphical abstract

Aerobic oxidation of methane over Pt/TiO_2_ in the presence of liquid water results in the formation of formaldehyde with a selectivity of 90 % at a methane conversion of up to 3 %.

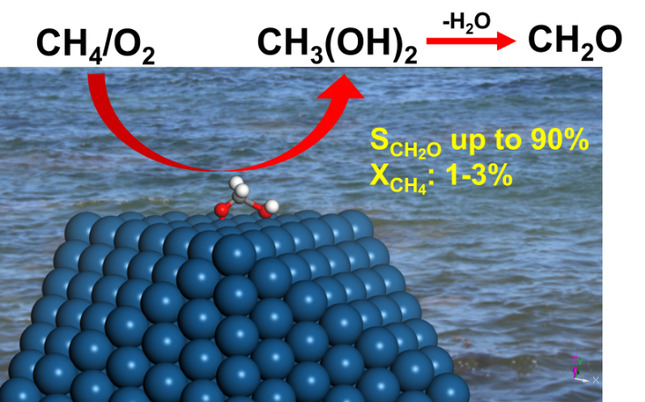

## Introduction

Methane is widely available in the form of natural gas, biogas or gas coming off landfill sites. It is being used for heating purposes, power generation or as a raw material for the chemical industry. The latter typically requires the transformation of methane into synthesis gas, a mixture of carbon monoxide and hydrogen, at temperatures higher than 800 °C, which can then be converted further. For instance, formaldehyde is produced by first passing synthesis gas over a Cu/ZnO‐catalyst yielding methanol in a high pressure, equilibrium‐limited reaction at temperatures between 250–300 °C, which is subsequently oxidised to formaldehyde at temperatures between 350–700 °C depending on the process of choice.^[1]^ The overall production of formaldehyde from methane has a relatively low exergy efficiency of only 43 %,^[2]^ with the greatest exergy losses associated with the formation of syngas. The direct, aerobic conversion of methane to formaldehyde would thus yield a more efficient process.

The challenge in the selective methane activation is often attributed to the limited reactivity of methane. However, enforcing formation of the desired product whilst at the same time limiting its consecutive conversion to CO_2_, is at least as challenging.^[3]^ For example, both the autocatalytic, homogeneous^[4]^ and heterogeneously catalysed^[5]^ oxidation of methane to formaldehyde show evidence of the fast consecutive oxidation of formaldehyde under those reaction conditions, thus requiring finetuning of the residence time to maximize its yield in these processes.

The consecutive reactions in the oxidation of methane can be minimized by using alternative oxidants and/or carrying out the reaction at relatively low temperatures. Some success in the selective oxidation of methane has been achieved using sulfuric acid,^[6]^ H_2_O_2_ ^[7]^ or N_2_O^[8]^ as the oxidant. However, the use of oxygen[^9^, ^10^, ^11^, ^12^, ^13^, ^14^, ^15^, ^16^, ^17^, ^18^, ^19^] or even air as the oxidant is necessary to make the process economically viable. It should be noted further that heat is an important side product in the selective methane oxidation (the oxidation of methane to formaldehyde releases 34 % of the lower heating value of methane), which will need to be recovered to obtain an energy‐efficient process. Effective heat removal requires operating at somewhat elevated temperatures, typically in the range of 200–300 °C to co‐generate medium to high pressure steam.

Water facilitates the selective oxidation of methane.[^9^, ^13^, ^14^, ^15^, ^16^, ^17^, ^20^] The mechanism for the observed enhancement may be system specific. The photo‐catalytic oxidation of methane oxidation over single Au‐atoms supported on black phosphorous nanosheets is thought to be accelerated in the presence of water through the generation of hydroxyl radicals.^[16]^ Rodriguez et al.[^9^, ^14^, ^15^] using inverse model catalysts (MeO/Cu_2_O/Cu(111) with Me=Ce, Zn) observed an improved selectivity for the formation of methanol in the conversion of methane upon co‐feeding water over these model catalysts. Co‐feeding water in the oxidation of methane oxidation did not only favour the formation of selective oxidation products, but also inhibited the formation of the complete oxidation products, CO_*x*_. This was ascribed to the generation of active surface hydroxyl species facilitating the formation of surface methoxy species at the metal‐oxide interface. The preferred formation of surface methoxy species over the formation of surface methyl species is necessary as the latter provides a pathway to CO_*x*_,[^13^, ^21^, ^22^] whereas the desorption of surface methoxy species could result in the formation of methanol.[^13^, ^21^]

Hence, the characteristics of catalysts for the selective oxidation of methane can be defined as follows; they should be able to dissociate the C−H bond in methane, operate at somewhat elevated temperature (preferably 200–300 °C), to utilize a cheap oxidant, such as oxygen or air, and to yield surface methoxy species rather than surface methyl species upon activation of methane. The methoxy species should adsorb not too strongly so that desorption as a selective oxidation product remains feasible.

Here, we explore the use of platinum for the selective oxidation of methane. The formation of surface methoxy species may become preferred over surfaces saturated with oxygen containing species.^[21]^ Full coverage of Pt(111) is difficult to achieve due to lateral interactions between adsorbed oxygen atoms^[23]^ and a maximum coverage of adsorbed oxygen of ca. 0.44 ML has been reported.^[24]^ It is, however, possible to increase the surface coverage with oxygen containing species by co‐adsorbing water.[^25^, ^26^, ^27^] Hence, we explore the role of water on the aerobic, selective oxidation of methane over supported catalysts containing 10 wt % platinum.

## Results and Discussion

**Figure 1:**
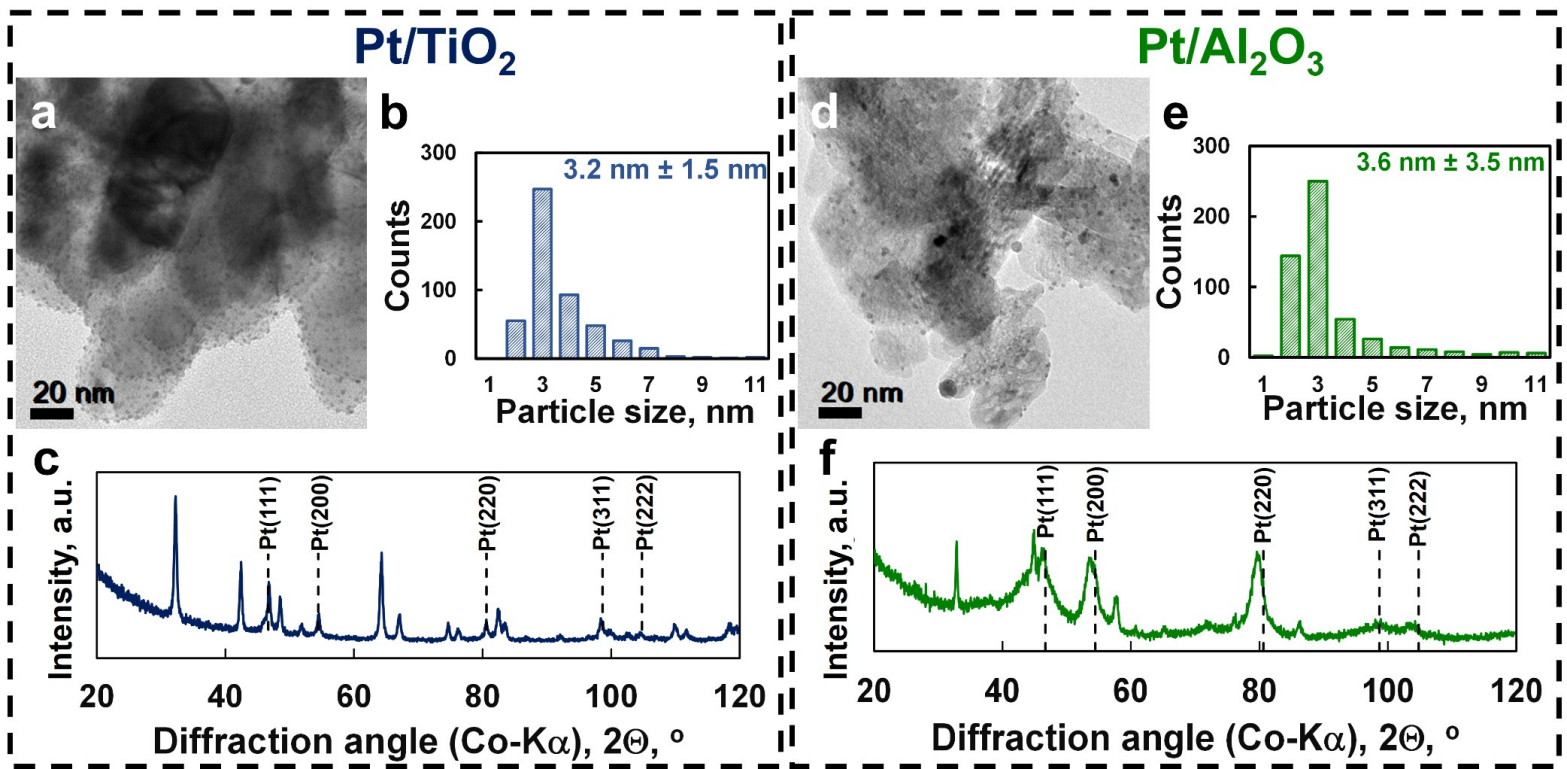
Transmission electron micrograph of the 10 wt % Pt/TiO_2_ catalyst (a) and the Pt/Al_2_O_3_ catalyst (d), particle size distribution of platinum on Pt/TiO_2_ (b) and Pt/Al_2_O_3_ (e), and powder X‐ray diffractogram of Pt/TiO_2_ (c) and Pt/Al_2_O_3_ (f) showing the presence of FCC−Pt.

The catalysts were prepared using incipient wetness impregnation of TiO_2_ (rutile) and γ‐Al_2_O_3_ with a platinic acid solution to obtain a metal loading of 10 wt.‐% (as confirmed using ICP‐OES—see Table S.1) in the reduced catalyst (*T*_red_=400°C, *t*_red_=5 hrs) Representative transmission electron microscopy bright field (TEM‐BF) images show spherical platinum nanoparticles, well‐dispersed on the support materials (Figure 1a and d). The particle size distributions were determined by measuring the size of 400 arbitrarily chosen nanoparticles on the images (Figure 1b and e). The average platinum particle size was 3.2±1.5 nm for Pt/TiO_2_ and 3.6±3.5 nm for Pt/Al_2_O_3_. This corresponds to an initial platinum dispersion of 41 % in Pt/TiO_2_ and 46 % in Pt/Al_2_O_3_, respectively, when considering the full particle size distribution. Powder X‐ray diffraction (PXRD) patterns show that platinum in both Pt/TiO_2_ (Figure 1c) and Pt/Al_2_O_3_ (Figure 1f) is present as metallic FCC‐platinum. The average crystalline domain size of platinum in Pt/Al_2_O_3_ was determined to be 4.4 nm in good agreement with the average platinum particle size determined from the TEM‐BF‐images. The average crystalline domain size of platinum in Pt/TiO_2_ could not be determined accurately due to the overlap of the diffraction lines.

The catalysts were initially tested in a fixed bed reactor set‐up with all components in the gas phase. The formation of interesting products, such as methoxy methanol and 1,3,5 trioxane, was observed when flooding the reactor (see Figures S.3 and S.4). Hence, a dedicated trickle bed reactor was constructed (see Figures S.1 and S.2) and the catalysts shown here were tested under continuous flow conditions for their activity and selectivity in the aerobic oxidation of methane in this set‐up at 493 K. Water was added to the feed whilst keeping the inlet partial pressure of methane and oxygen constant at 0.5 bar and 1.5 bar, respectively. Typical runs lasted several hundreds of hours regularly returning to standard conditions (Figures S.5 and S.7).

**Figure 2:**
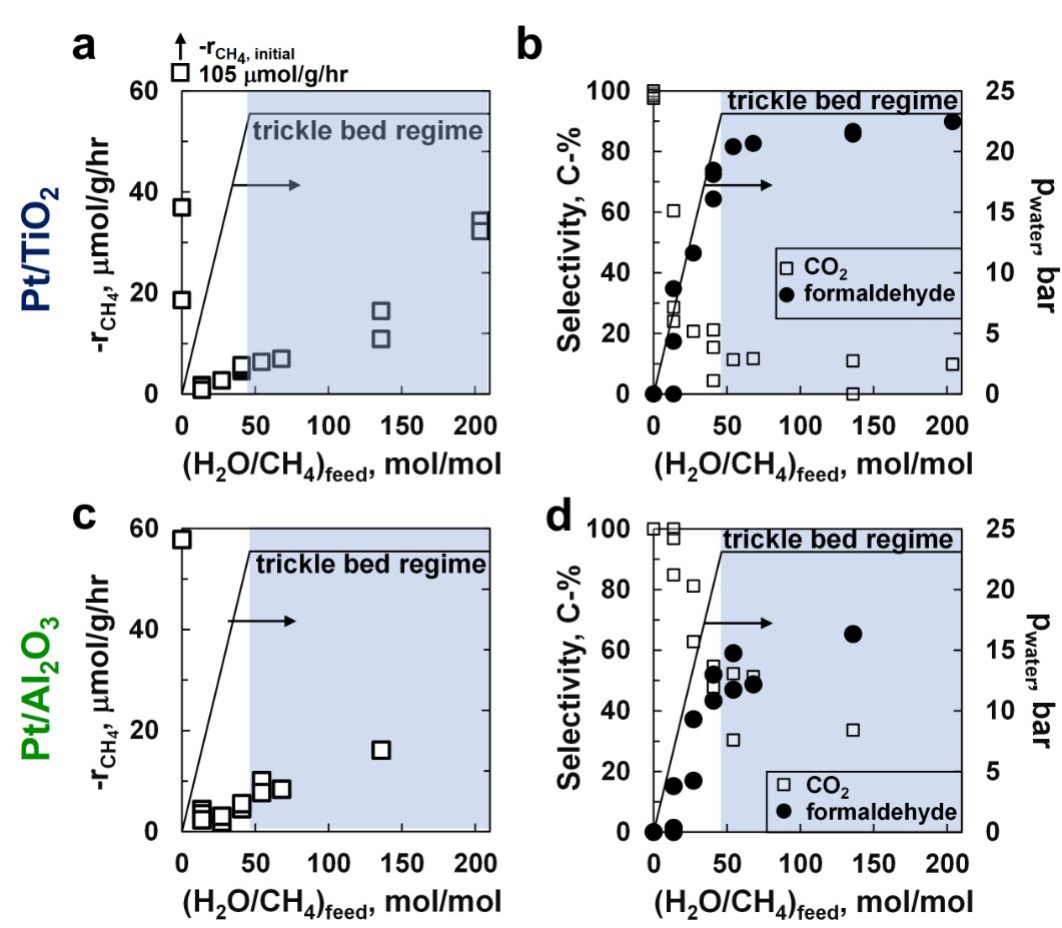
The rate of methane consumption over Pt/TiO_2_ (a) and Pt/Al_2_O_3_ as a function of water partial pressure (c), and the corresponding selectivity for the formation of formaldehyde and CO_2_ over Pt/TiO_2_ (b) and Pt/Al_2_O_3_ (d). *T*_reaction_=493 K, *p* ${}_{\mathrm{CH}{}_{4}}$_, inlet_=0.5 bar, *p* ${}_{O{}_{2}}$_, inlet_=1.5 bar, F${}_{\mathrm{CH}{}_{4},0}$ /W=3.23 mmol g^−1^ h^−1^.

Figure 2a–d shows the steady‐state activity and selectivity in the oxidation of methane over the reduced platinum catalysts at 493 K as a function of the molar inlet ratio of water to methane. The introduction of water in the feed results in an initial 10 to 50‐fold drop in the rate of methane consumption at steady‐state. The drop in the catalytic activity was expected as the surface becomes increasingly more covered with adsorbed water and surface hydroxyl species,[^25^, ^26^, ^27^] thus reducing the likelihood of direct interaction of methane with the bare platinum surface. Increasing the ratio of water to methane in the feed further, but keeping the fluid phase as a gas phase, has a positive effect on the catalyst activity.

Interestingly, the rate of methane oxidation starts to increase more rapidly upon entering the trickle‐bed regime i.e., with water now present as a liquid in the catalyst bed, which is observed at an inlet molar ratio of H_2_O/CH_4_ higher than 46 under the applied conditions. An activity of 35 μmol g^−1^_cat_ h^−1^ was obtained over Pt/TiO_2_ with a feed ratio of H_2_O/CH_4_=204, which corresponds to a turnover frequency of 0.17 h^−1^ assuming a dispersion of platinum of 41 % (deduced from the particle size distribution in Figure 1b). The obtained maximum rate of methane oxidation over Pt/Al_2_O_3_ was lower at 16 μmol g^−1^_cat_ h^−1^ (albeit at a lower inlet molar ratio of H_2_O/CH_4_ of 136). The role of water in the aerobic, selective oxidation of methane was highlighted previously,[^9^, ^13^, ^14^, ^15^, ^16^, ^17^, ^20^] but here we show unequivocally the need to perform the reaction in the presence of liquid water to accelerate the aerobic oxidation of methane. The increase in the activity upon entering the trickle bed regime changes gradually, possibly due to the incomplete wetting of the catalyst by liquid water.^[28]^ The wetting efficiency (i.e., the fraction of surface covered by liquid water) increases with increasing superficial mass velocity of the liquid, but under the applied reaction conditions the wetting efficiency is estimated^[29]^ to be less than 40 % at the highest employed inlet molar ratio of H_2_O/CH_4_.

The observed 3–20 times increase in the rate of methane oxidation upon partial wetting of the catalyst is surprising. The fugacity of water remains constant when moving into the trickle bed regime and thus the fraction of the surface covered with adsorbed water and species such as OH and O is not expected to change if these surface species are equilibrated with oxygen and water in the fluid phase. Various explanations for the observed strong increase in the rate of methane oxidation upon partial wetting the catalyst can be put forward, viz. a change in the stability of the adsorbates through interaction with the liquid water,^[30]^ solvation effect of the product,^[20]^ or a kinetic effect if water is involved in the rate controlling step (wetting of the surface under the applied conditions results in a 100‐fold increase in the concentration of water near the catalytically active site). At this point no firm conclusions regarding the exact origin of this phenomenon can be made.

Increasing the water content in the feed induces a change in the product selectivity from forming only CO_2_ in the absence of water to the selective formation of formaldehyde, with a selectivity of ca. 90 C‐% when maintaining a H_2_O/CH_4_ ratio of ca. 100–200 in the feed over Pt/TiO_2_. The high selectivity for the formation of formaldehyde is obtained before entering the trickle bed regime hinting at a decoupling between catalytic activity and selectivity.

**Figure 3:**
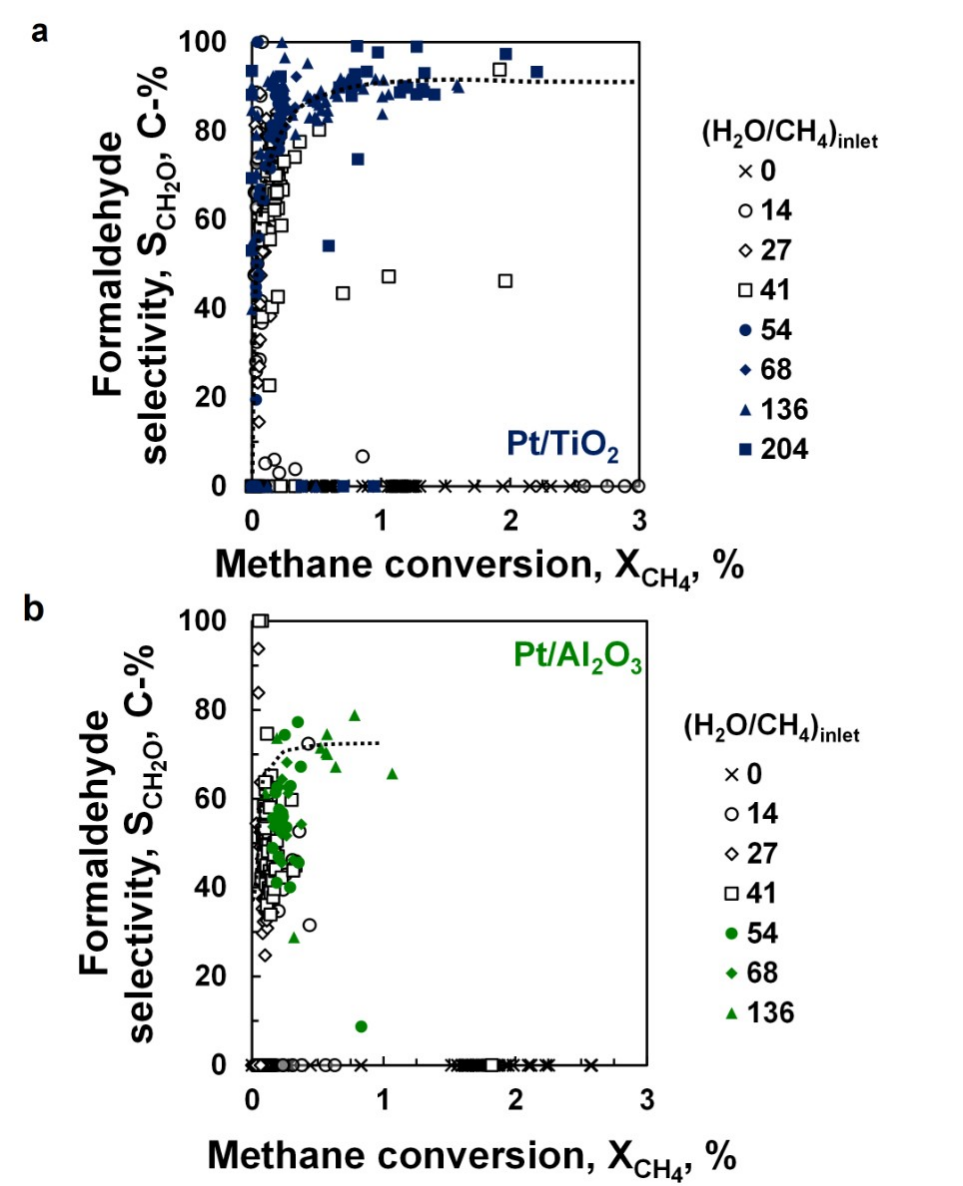
Selectivity of formaldehyde as a function of the methane conversion over Pt/TiO_2_ (a) and Pt/Al_2_O_3_ (b) (x: without water in the feed; open symbols: gas phase reaction; solid symbols: trickle bed conditions). Operating conditions: *T*=493 K, *p* ${}_{\mathrm{CH}{}_{4}}$ =0.5 bar, *p* ${}_{O{}_{2}}$ =1.5 bar, F${}_{\mathrm{CH}{}_{4},0}$ /W=3.2 mmol g^−1^ h^−1^.

The selective oxidation of methane often suffers from the so‐called selectivity‐conversion limitation[^20^, ^31^] wherein the selectivity of the desired selective oxidation product drops, often sharply, with increasing conversion. This can be rationalised by considering a common surface intermediate leading to the formation of the selective oxidation product and CO_*x*_. Re‐adsorption of the selective oxidation product will then result in enhanced formation of CO_*x*_. A high rate for the consecutive oxidation of the common surface intermediate relative to the net rate of desorption of the selective oxidation product will result in a sharp drop in the selectivity for the formation of the selective oxidation product upon increasing the conversion of methane. Limiting the rate for the consecutive reactions leading to the formation of CO_*x*_ relative to the net rate of desorption should ultimately yield an (almost) constant selectivity for the selective oxidation product as a function of the conversion. Transforming the obtained data at different inlet ratios of water to methane (Figures S.5 and S.7) into a plot of the formaldehyde selectivity as a function of the methane conversion (Figure 3) shows that the selectivity for the formation of formaldehyde is almost constant at a high (H_2_O/CH_4_)_feed_ (>50) and does not decrease with increasing conversion (up to X${}_{\mathrm{CH}{}_{4}}$ ≈3% for Pt/TiO_2_). The observed increase in the selectivity for the formation of formaldehyde with increasing conversion at lower conversion levels is attributed to the different kinetic regimes, to which the catalyst was exposed at the lower inlet ratios of methane to water.

The formaldehyde selectivity over Pt/Al_2_O_3_ appears to be less than over Pt/TiO_2_. Primarily formed formaldehyde can be further converted to CO_2_, in particular over basic sites as present in boehmite,^[32]^ to which γ‐Al_2_O_3_ transforms upon exposure to hydrothermal conditions at this temperature (vide infra). The conversion of formaldehyde may proceed directly or a Cannizzaro‐type of reaction involving formaldehyde and water (and indeed some methanol is sometimes observed under these conditions).

**Figure 4:**
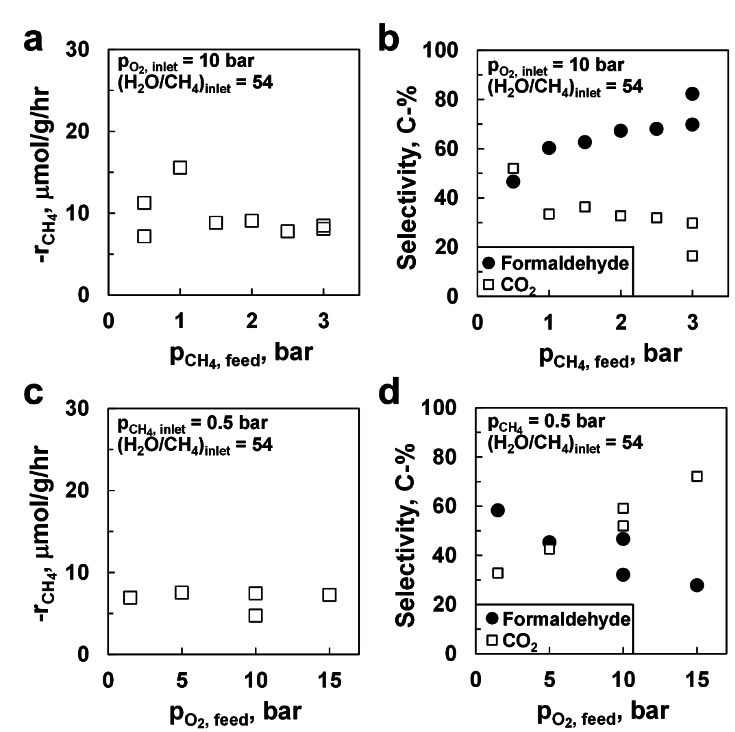
The effect of the methane partial pressure (*p* ${}_{O{}_{2}}$_,inlet_=10 bar) on the rate of methane oxidation (a) and formaldehyde selectivity (b) over Pt/Al_2_O_3_, and the effect of the oxygen partial pressure (*p* ${}_{\mathrm{CH}{}_{4}}$_,inlet_=0.5 bar) on the rate of methane oxidation (c) and formaldehyde selectivity (d) over Pt/Al_2_O_3_ under trickle bed conditions at 493 K, *p* ${}_{H{}_{2}O}$ =23.1 bar, (H_2_O/CH_4_)_feed_=54 and F${}_{\mathrm{CH}{}_{4},0}$ /W=3.2 mmol g^−1^ h^−1^.

The kinetics of the selective oxidation of methane was further probed under trickle bed conditions (H_2_O/CH_4_=54) in a separate run starting with freshly reduced Pt/Al_2_O_3_ catalyst. The rate of methane consumption is independent of the partial pressure of methane (Figure 4a). A first order dependency is expected if the reaction is limited by methane activation; a rate of reaction independent of the methane partial pressure indicates that the rate determining step involves species whose surface concentrations are hardly affected by the change in the methane partial pressure (e.g., because their surface concentration is at its saturation level).

The rate of oxidation of methane under trickle bed conditions over Pt/Al_2_O_3_ is also independent of the oxygen partial pressure (Figure 4c). This implies that the partial pressure of oxygen does not affect the surface coverage of the species involved in the rate determining step.

The observed kinetic dependencies are consistent with the desorption of the selective oxidation product being the rate controlling step in the aerobic oxidation of methane over Pt/Al_2_O_3_ under the applied conditions.

The selectivity for the formation of formaldehyde over Pt/Al_2_O_3_ increases first strongly and then gradually with increasing partial pressure of methane (Figure 4b). A selectivity of 69 % for the formation of formaldehyde was observed when starting up the reaction and exposing the catalyst to a feed with *p* ${}_{\mathrm{CH}{}_{4}}$ =3 bar (and *p* ${}_{O{}_{2}}$ =10 bar; (H_2_O/CH_4_)_feed_=54). Subsequently, the methane partial pressure was dropped to 0.5 bar (keeping the partial pressure of oxygen and the feed ratio of water to methane constant) resulting in a drop in the selectivity of formation of formaldehyde to 47 % (the lower selectivity for the formation of formaldehyde at these inlet conditions was verified in an independent measurement). The inlet partial pressure of methane was subsequently increased stepwise resulting in an increase in the selectivity for the formation of formaldehyde. Returning to an inlet partial pressure of methane of 3 bar yielded a selectivity for the formation of formaldehyde of 82 % over this catalyst. This implies that the selectivity may not only be affected by the change in the partial pressure of methane but also by changes in the catalyst, although this does not affect the catalyst activity.

The selectivity for the formation of formaldehyde decreases with increasing partial pressure of oxygen (Figure 4d). This may be attributed to a secondary conversion of primarily formed formaldehyde to CO_*x*_.[^31^, ^32^, ^33^]

**Figure 5:**
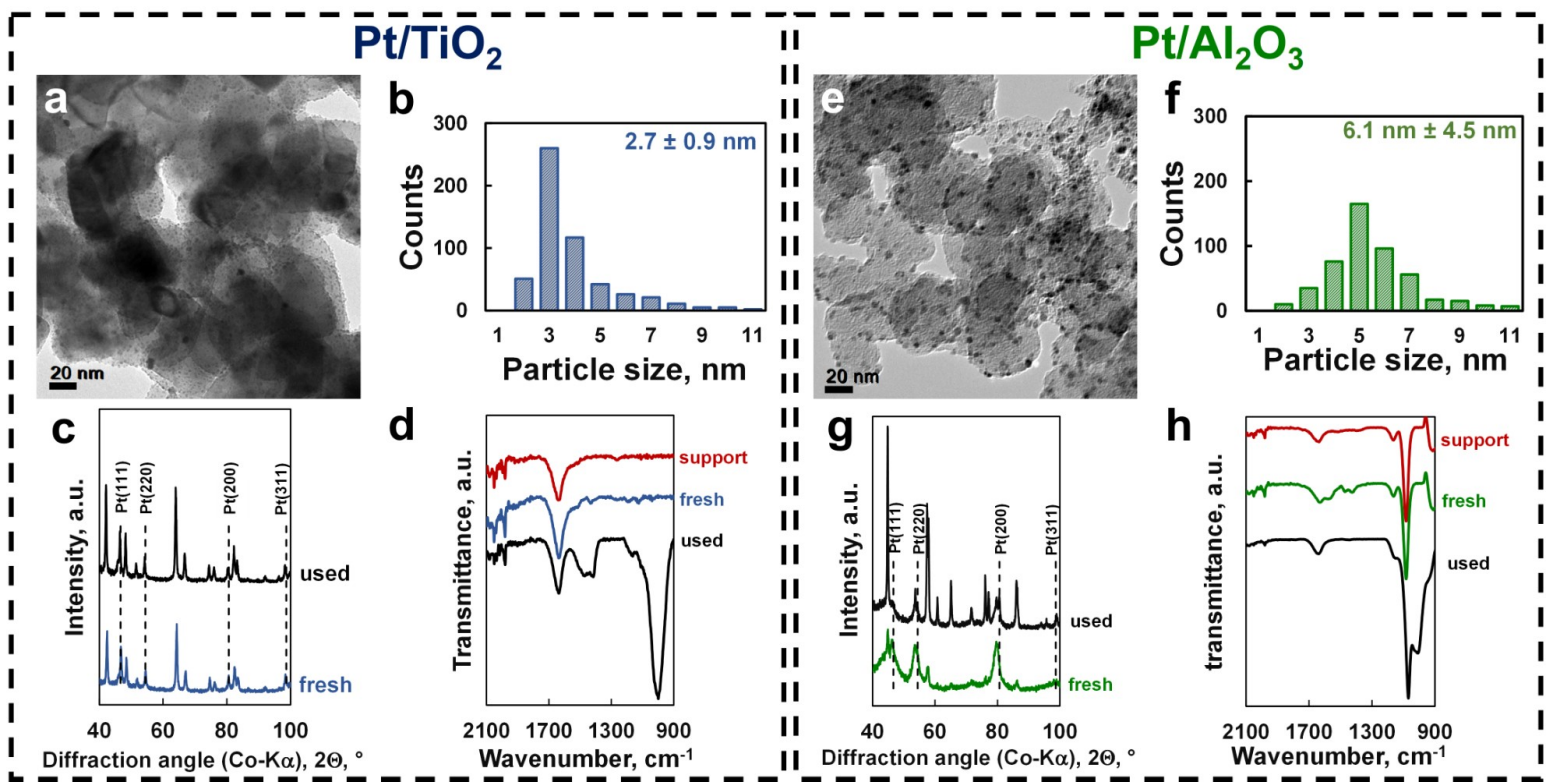
Transmission electron micrograph of the used 10 wt % Pt/TiO_2_ catalyst (a) and the Pt/Al_2_O_3_ catalyst (e), particle size distribution of platinum on Pt/TiO_2_ (b) and Pt/Al_2_O_3_ (f), powder X‐ray diffractogram of Pt/TiO_2_ (c) and Pt/Al_2_O_3_ (g) before and after exposure to the hydrothermal condition of the selective, aerobic oxidation of methane, and the ATR‐FTIR spectra of the support and the samples before and after testing Pt/TiO_2_ (d) and Pt/Al_2_O_3_ (h) in the selective oxidation of methane.

The catalysts showed signs of deactivation (Figures S.5, S.7, S.8), as indicated by activity loss during periodic returns to operating conditions in the absence of water and/or an inlet molar ratio of water to methane in the feed of 14. The platinum particle size distribution in the Pt/TiO_2_ catalyst after exposing it to the strong hydrothermal conditions of methane oxidation for more than 300 hrs was almost unchanged (Figure 5a) and a platinum dispersion of 39 % was determined (based on the whole particle size distribution in Figure 5b). The powder X‐ray diffraction pattern (PXRD) of Pt/TiO_2_ shows that the titania support remained present in the rutile phase and platinum as FCC−Pt (Figure 5c). The attenuated total reflectance‐infrared (ATR‐IR) spectrum of the used catalyst shows the appearance of a new strong absorption band at 999 cm^−1^ (Figure 5d), which is attributed to C−O−C stretching in polyoxymethylene with a low degree of polymerization or paraformaldehyde.[^34^, ^35^] The absorption bands at 1417 cm^−1^ and 1472 cm^−1^ are indicative of the C−H bending mode in polyoxymethylene.^[36]^ Deposition of the oligomers of formaldehyde or polyoxymethylene on Pt/TiO_2_ may thus be the cause of the observed catalyst deactivation in this catalyst.

Large changes are observed in the Pt/Al_2_O_3_ catalyst after exposure to the hydrothermal conditions of selective oxidation of methane (Figure 5e–h). The platinum dispersion in the used Pt/Al_2_O_3_ catalyst decreases from 46 % to 24 %. Furthermore, the alumina support in the catalyst has undergone a phase transition and is present mainly as boehmite in the used catalyst (Figure 5g). Platinum is still present as FCC−Pt. The phase transition of γ‐Al_2_O_3_ to boehmite under the applied hydrothermal conditions is rapid[^37^, ^38^] and is typically associated with a loss in the surface area of the support as well sintering of the deposited platinum particles,^[37]^ and thus a loss in the metal surface area. The metal loading of the used catalyst indicated that leaching of platinum did not occur. The ATR‐FTIR spectrum shows again the strong absorption band at 1000 cm^−1^, but the characteristic bands at ca. 1420 and 1470 cm^−1^ indicative of the C−H bending mode in polyoxymethylene^[36]^ are absent. The appearance of the band at 1000 cm^−1^ may be related to the structural change in the support when exposing the catalysts to liquid water under reaction conditions. The decrease in catalyst activity seen over Pt/Al_2_O_3_ is (at least partly) attributed to the change in the platinum metal surface area.

Density functional theory (DFT) calculations were performed to further investigate the preferential formation of formaldehyde over platinum‐based catalysts. We focused on the Pt(111) surface as this surface is expected to be dominant on platinum nano‐particles.[^39^, ^40^] Furthermore, oxygen‐containing species are bonded weaker on Pt(111) than on Pt(100),^[41]^ and product compounds adsorbed on Pt(111) may desorb more readily.

First, we explored the phase space of the coverages with atomic oxygen, surface hydroxyl groups and adsorbed water on a (√3×√3)‐Pt(111), a (2×2)‐Pt(111) and a (3×3)‐Pt(111) surface using ab initio thermodynamics^[42]^ to map out conditions at which the surface could be fully covered with oxygen containing species (the selective oxidation of methane is thought to be facilitated when the surface is fully covered with oxygen containing species).^[21]^ Many different possible configurations have been probed (Table S.2). This map gives thus a reasonable indication of the favourable reaction conditions for the selective aerobic oxidation of methane.

**Figure 6:**
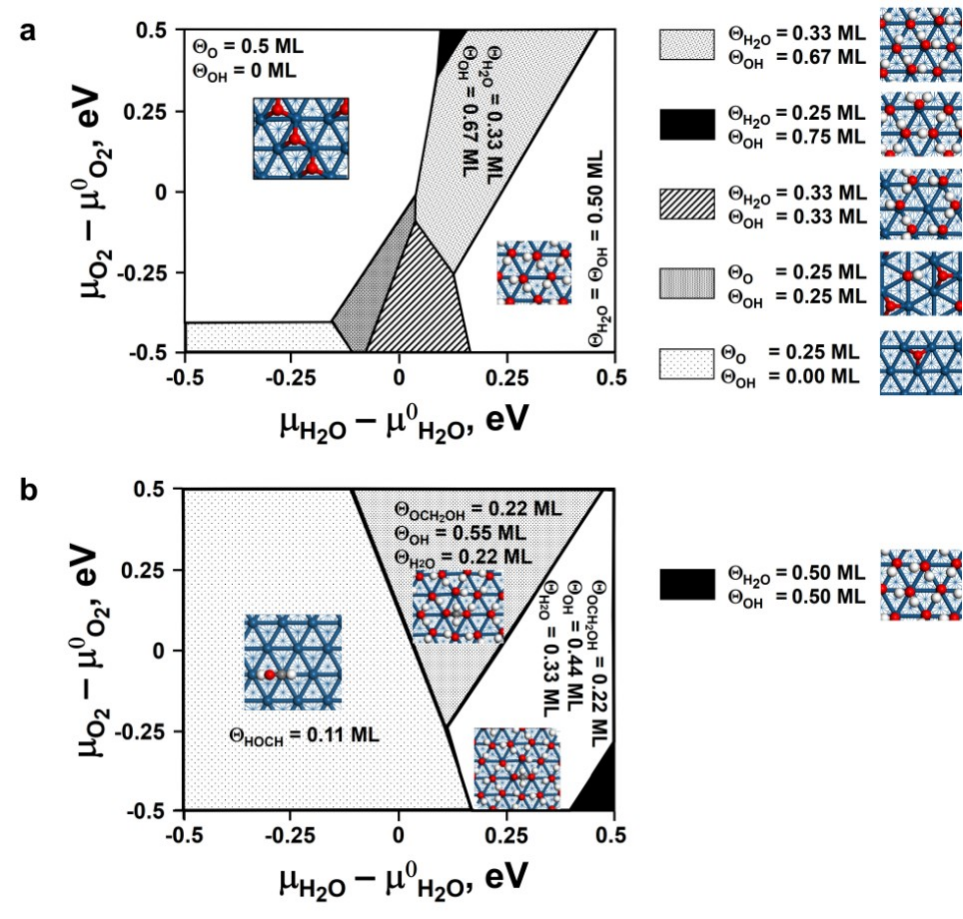
Surface phase diagram upon adsorption of H_2_O and O_2_ (a) and H_2_O, O_2_ in the presence of CH_4_ (*p* ${}_{\mathrm{CH}{}_{4}}$ =0.5 bar) (b) at 493 K on Pt(111) (chemical potential of O_2_ and H_2_O relative to their chemical potential at 493 K and 1 bar;—note water will condense at *μ* ${}_{H{}_{2}O}$ −*μ* ${}_{H{}_{2}O}$ ^0^=0.133 eV and 493 K; hydrogen: white; oxygen: red; surface platinum: dark cyan).

At a low chemical potential of water (corresponding to a low partial pressure of water), the coverage of Pt(111) with oxygen is limited to ca. 0.50 ML (Figure 6a), a value in good agreement with the experimental saturation coverage with oxygen of 0.44 ML.^[24]^ Higher surface coverage may be obtained by exposing the platinum surface to a mixture of water and oxygen as surface hydroxyl species can form a so‐called honeycomb structure on the surface,[^25^, ^26^, ^27^] in which 2/3 of the surface is covered with adsorbate. Surface coverages of 80–89 % have been reported experimentally^[27]^ and they become thermodynamically stable at higher chemical potential of water and oxygen (i.e., at elevated partial pressures). For instance, a structure containing 0.33 ML H_2_O and 0.67 ML OH (Figure 6a) was determined to be the most stable structure at 493 K and a chemical potential of oxygen of 0 eV (relative to the chemical potential of oxygen at 493 K and 1 bar) and a chemical potential of water of 0.04 eV (relative to the chemical potential of water at 493 K and 1 bar), which corresponds to a partial pressure of oxygen and water of 1 and 2.6 bar, respectively. In this structure, all surface platinum atoms on Pt(111) are covered with adsorbate species. The structure containing a coverage of 0.50 ML H_2_O and 0.50 ML OH (as indicated in Figure 6a) will not exist as water condenses out at a chemical potential of 0.133 eV relative to the chemical potential of water at 1 bar and 493 K.

The activation of methane on Pt(111) partially covered with adsorbed atomic oxygen may result in the formation of a surface methyl species, a surface methoxy species, adsorbed di‐σ formaldehyde^[43]^ or further decomposition products.^[44]^ At a low chemical potential of oxygen and water and a partial pressure of methane of 0.5 bar (Figure 6b), a surface hydroxy methylene species is the most stable intermediate on Pt(111). Hydroxy methylene does not desorb as formaldehyde but rather decomposes to CO.^[44]^

The interaction of methane with Pt(111) saturated with adsorbed H_2_O and OH species, which is obtained at higher chemical potential of water, favours the formation of a very stable di‐σ hydroxy‐methoxy species. Upon increasing the oxygen partial pressure, this intermediate becomes the most stable species at a lower partial pressure of water and may play an important role in the selective oxidation of methane.

**Scheme 1:**
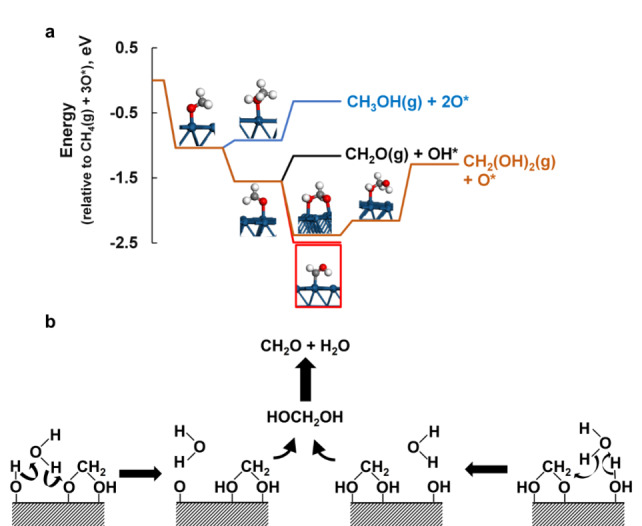
Possible reaction pathway for product formation over Pt(111) in the selective oxidation of methane (a) and role of water as a H‐transferring shuttle in the formation of methanediol (b).

We observe the direct formation of formaldehyde with high selectivity (up to 90 C‐% at a methane conversion of up to 3 % over Pt/TiO_2_) in the selective oxidation of methane over platinum A pathway leading to the formation of methanol and formaldehyde can be easily established over a bare Pt(111) surface (Scheme 1a), if the activation of methane results in the formation of a surface methoxy species.[^13^, ^21^] Hydrogen transfer from either co‐adsorbed water or surface hydroxyl species to the surface methoxy species would yield adsorbed methanol, and upon desorption yield methanol in the gas phase. Hydrogen transfer from the surface methoxy species to co‐adsorbed hydroxyl species could result in the formation of adsorbed formaldehyde, CH_2_O*, which could desorb resulting in the formation of formaldehyde is the gas phase. However, this reaction pathway cannot explain readily the preferred formation of formaldehyde over methanol as observed experimentally.

The addition of a surface hydroxyl species to adsorbed formaldehyde may result in the formation of the stable di‐σ‐hydroxy‐methoxy species. Hydrogen transfer from co‐adsorbed water or surface hydroxyl species would result in the formation of adsorbed methanediol, which would desorb as the hydrated form of formaldehyde, methanediol.^[45]^ The formation of the strongly adsorbed di‐σ‐hydroxy‐methoxy species may explain the observed high selectivity for the formation of formaldehyde in the absence of formation of methanol. It may further result in the desorption (rather than methane activation) becoming the rate controlling step, in accordance with the observed zero order dependency of the rate of reaction with respect to the partial pressure of methane and oxygen.

The presence of liquid water in the selective oxidation of methane over Pt/TiO_2_ and Pt/Al_2_O_3_ has been shown to enhance the rate of reaction. Various thermodynamic reasons for the observed enhancement have been put forward;[^20^, ^30^] water may also have a kinetic effect, assisting in the hydrogen transfer between adsorbed surface intermediates, thereby enhancing product desorption (see Scheme 1b). Water will interact with adsorbed surface hydroxyl species, adsorbed water, and other surface species through H‐bonding. Water in the liquid phase can orientate itself towards the surface species, thus maximizing its interaction with various surface species. Hydrogen transfer from a surface hydroxyl species or adsorbed water to a di‐σ‐hydroxy‐methoxy species could be facilitated by a water molecule in the fluid phase interacting with both species, simultaneously accepting hydrogen from either an adsorbed hydroxyl species or adsorbed water and donating it to the di‐σ‐hydroxy‐methoxy intermediate in a concerted reaction, which upon desorption would yield methanediol in the gas phase.

Methanediol can undergo various reactions in the reactor (and even in the reactor effluent). It may dehydrate resulting in the formation of formaldehyde,^[45]^ although its homogeneous uni‐molecular decomposition rate is less than 2×10^−6^ s^−1^ ^[46]^ (but it may be facilitated by wall material). It may also polymerise/oligomerise resulting in catalyst deactivation (as evidence for polyoxymethylene was observed in the used Pt/TiO_2_ catalyst).

## Conclusion

The selective, aerobic oxidation of methane over platinum‐based catalysts results in the selective formation of formaldehyde. The preferential formation of formaldehyde is thought to proceed via the strongly adsorbing di σ‐hydroxy methoxy species on Pt(111) which would result in the formation of formaldehyde in its hydrated form, methanediol. The rate of reaction is independent of the partial pressure of methane and oxygen, but strongly affected by the presence of water in the feed. The reaction is further enhanced when the catalyst is in contact with liquid water. Water is thought not only to saturate the surface thus minimizing the presence of precursors for the formation of CO_*x*_, but also to facilitate the desorption of the di σ‐hydroxy methoxy intermediate yielding methanediol (formaldehyde hydrate) and formaldehyde.

Improved catalysts may be obtained by weakening the adsorption of the di σ‐hydroxy‐methoxy species, facilitating the desorption from the platinum surface as the desired product and reducing the extent of polymerisation on the surface. This may be achieved by alloying the catalytically active metal and altering the change in the strength of adsorption by changing the d‐band centre.

## Conflict of interest

The data in this manuscript form part of the patent application GB2115453.9

1

## Supporting information

As a service to our authors and readers, this journal provides supporting information supplied by the authors. Such materials are peer reviewed and may be re‐organized for online delivery, but are not copy‐edited or typeset. Technical support issues arising from supporting information (other than missing files) should be addressed to the authors.

Supporting Information

## Data Availability

The data that support the findings of this study are available from the corresponding author upon reasonable request.
