# Supplementary material for: Platinum‐Catalysed Selective Aerobic Oxidation of Methane to Formaldehyde in the Presence of Liquid Water
Source: Angew Chem Int Ed Engl. 2022 Aug 16;61(38):e202206841. doi: 10.1002/anie.202206841 (PMC9541881; doi:10.1002/anie.202206841)
Supplement: Supplementary file 1 — Supporting Information [file ANIE-61-0-s001.pdf]

Angewandte Chemie
Eine Zeitschrift der Gesellschaft Deutscher Chemiker

Supporting Information

# Platinum-Catalysed Selective Aerobic Oxidation of Methane to Formaldehyde in the Presence of Liquid Water

*S. V. L. Mahlaba, N. Hytoolakhan Lal Mahomed, A. Govender, J. Guo, G. M. Leteba, P. L. Cilliers, E. van Steen**

Supplementary material to

# Platinum Catalysed Selective Aerobic Oxidation of Methane to Formaldehyde in the Presence of Liquid Water

by S.V.L. Mahlaba, N. Hytoolakhan Lal Mahomed, A. Govender, J. Guo, G.M. Leteba, P.L. Cilliers and E. van Steen

**Content:**

**Section I: Catalyst synthesis and characterization**

**Section II: Catalytic testing**

**Section III: DFT Studies**

## Section I: Catalyst synthesis and characterization

The catalysts were prepared by incipient wetness impregnation of platinic acid in deionized water on titanium dioxide (Sigma Aldrich; Rutile; $S_{BET}$ = 44.5 $m^2/g$), or alumina ($\gamma$-$Al_2O_3$; Alfa Aesar, 3 Micron APD Powder, LOT: K27Y013, $S_{BET}$ = 73.9 $m^2/g$) as a support. The support was contacted with an aqueous solution of platinic acid ($H_2PtCl_6$, Sigma Aldrich) to obtain 10 wt.-% platinum on the support. The solid was dried, calcined at 400°C (air flow rate: 48 $ml_n$/min/g) and subsequently reduced for 5 hrs at 400°C in flowing hydrogen (48 $ml_n$/min/g).

The elemental composition of the materials was determined using inductively coupled plasma-optical emission spectrometry (ICP-OES). The nanoparticles were imaged using transmission electron microscopy (TEM; FEI Tecnai G2 T20 TEM, operating at 200 kV). Specimens for TEM analysis were prepared by casting one-drop of a colloidal suspension in acetone onto 3-mm carbon-coated copper grids. These were then air dried under ambient conditions. The particle size distribution and the average particle size of the nanosized particles representing platinum was determined by measuring between 400-550 nanoparticles using ImageJ® software.

The phase composition of the catalyst samples was determined using powder X-ray diffraction (XRD) on a Bruker D8 ADVANCE diffractometer (Co-Kα radiation: λ = 1.789 Å, 35 kV, 40mA). The spent catalysts were characterized using Fourier transform infrared spectroscopy (FTIR) on a Perkin Elmer Spectrum 100 FTIR Spectrometer in the range 650-4000 $cm^{-1}$ with a resolution of 1 $cm^{-1}$.

**Table S.1:** Metal loading and dispersion of Pt-based catalysts (values in brackets for the used catalyst)

| **Sample** | **Pt/$Al_2O_3$** | **Pt/$TiO_2$(rutile)** |
|---|---|---|
| Pt-loading | 10.2 | 10.3 (10.2) |
| $T_{reduction}$, °C | 400 | 400 |
| $t_{reduction}$, hrs | 5 | 5 |
| $H_2$-uptake, $cm^3$(STP)/g | 1.19 | 0.81 (0.90) |
| Dispersion[a] | 20.6 | 14.3 (15.7) |
| $d_{Pt}$[b], nm | 5.5 | 7.9 (7.2) |
| $d_{Pt}$[c], nm | 3.2 ± 1.5 (3.6 ± 3.5) | 2.1 ± 0.9 (6.1 ± 4.5) |

[a]: based on $H_2$-chemisorption; [b]: estimated from $d_{Pt} = \frac{113}{Dispersion(\%)}$; [c]: from TEM-measurement

**Section II: Catalyst testing**

Figure S.1 shows the process flow diagram of the trickle bed reactor, whilst Figure S.2 shows the cross-sectional schematic drawing of the reactor tube. The reactor consisted of a quartz tube (length:38 cm; I.D.=12 mm packed with ca. 1.5 g of the pelletized and sieved catalyst (100-150 µm) in its centre. The void space on top of the catalyst was packed with silicon carbide particles ($d_p$ ~300µm, obtained from Colbern Abrasives cc, Parow, South Africa). The catalyst and the silicon carbide were held in place with 2 small glass wool plugs on either end. The quartz tube was placed inside a 19.05 mm O.D. stainless steel tube. A quartz-sheathed thermocouple was placed at the centre of the catalyst bed for temperature measurement. The reactor is enclosed in an aluminium heating furnace controlled with multiple heating zones each controlled by thermocouples on the outside of the reactor. The isothermal zone in the reactor was ca. 10 cm. Argon flowed pressure-controlled through the annular space between the two tubes at the same pressure as inside the quartz tubing. The bottom of the reactor rested on a bed of silicon carbide ($d_p$ ~ 300 µm; bed length 15.5 cm) to ensure evaporation of the liquid dripping out of the catalyst bed (the argon flow rate was typically set at ca. 2-3 times the total flow rate, including steam, through the catalyst bed). The temperature of the bottom zone was adjusted depending on the water flow rate to achieve smooth operation.

The reactor effluent passed a heated expansion valve placed directly under the reactor to reduce the pressure to atmospheric pressure and was transferred to the on-line GC ($T_{transfer\ line}$ = 180$^o$C), where it was injected over a heated 6-way valve. The effluent passed a condenser (operating at room temperature) and was vented into the vent line of the walk-in fume hood.

The products were analysed on a GC-FID (Agilent 6890N) equipped with a two-step methanation reactor (PolyArc$^{TM}$, Activated Research Company) and an FID. The Polyarc™ reactor is designed to enable calibration free analysis of all carbon containing compounds via an FID by first oxidizing the organic samples eluting out of a GC column with air to carbon dioxide and then reducing the carbon dioxide with hydrogen to methane before passing it onto the FID [S1]. The products were separated on a HP-PLOT Q PT capillary column (0.32 mm diameter, 30 m length, 20 µm stationary phase film thickness polystyrene-divinylbenzene; Agilent Technologies).

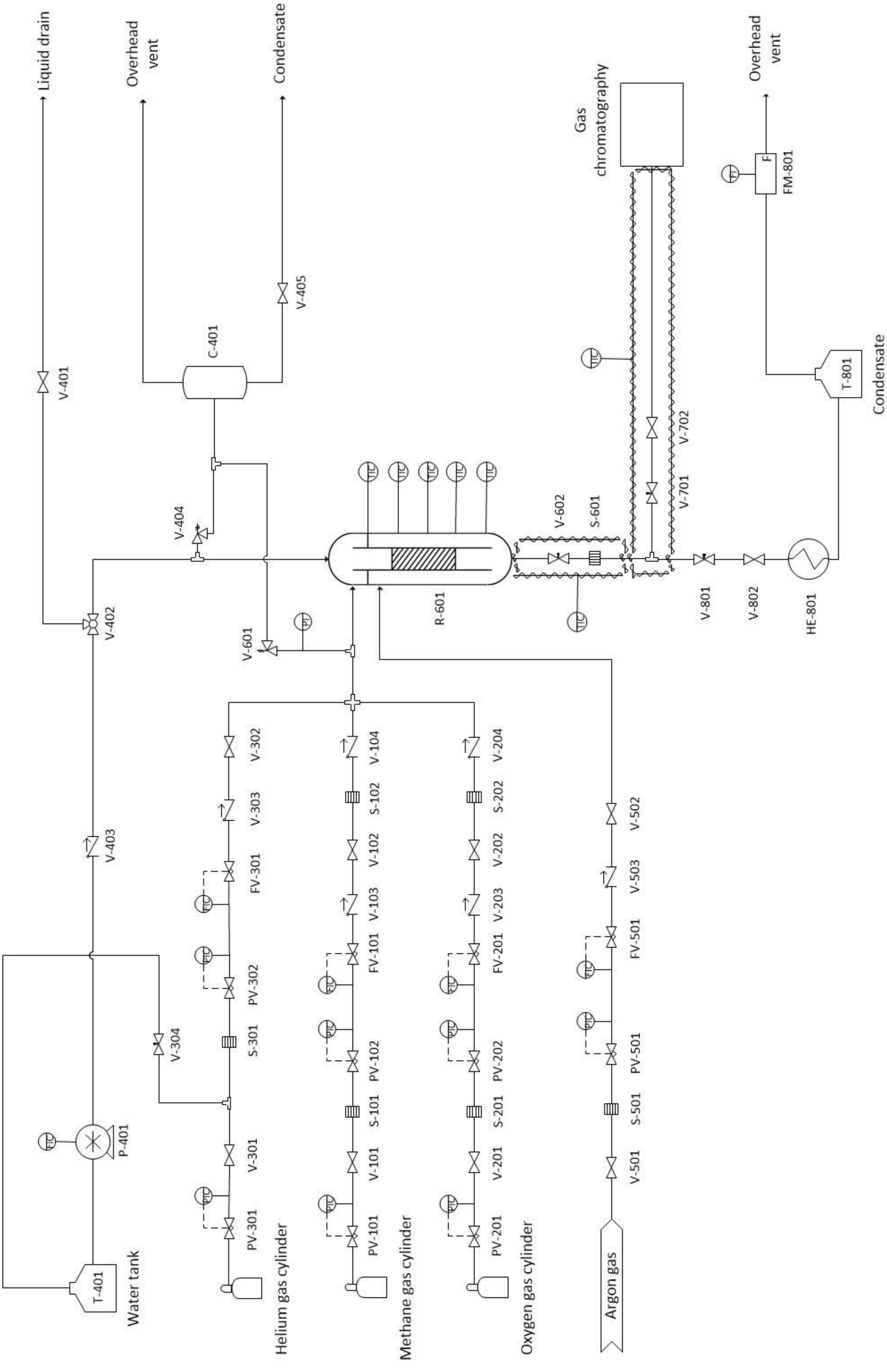


**Figure S.1**: Process flow diagram of the trickle-bed reactor for the selective oxidation of methane

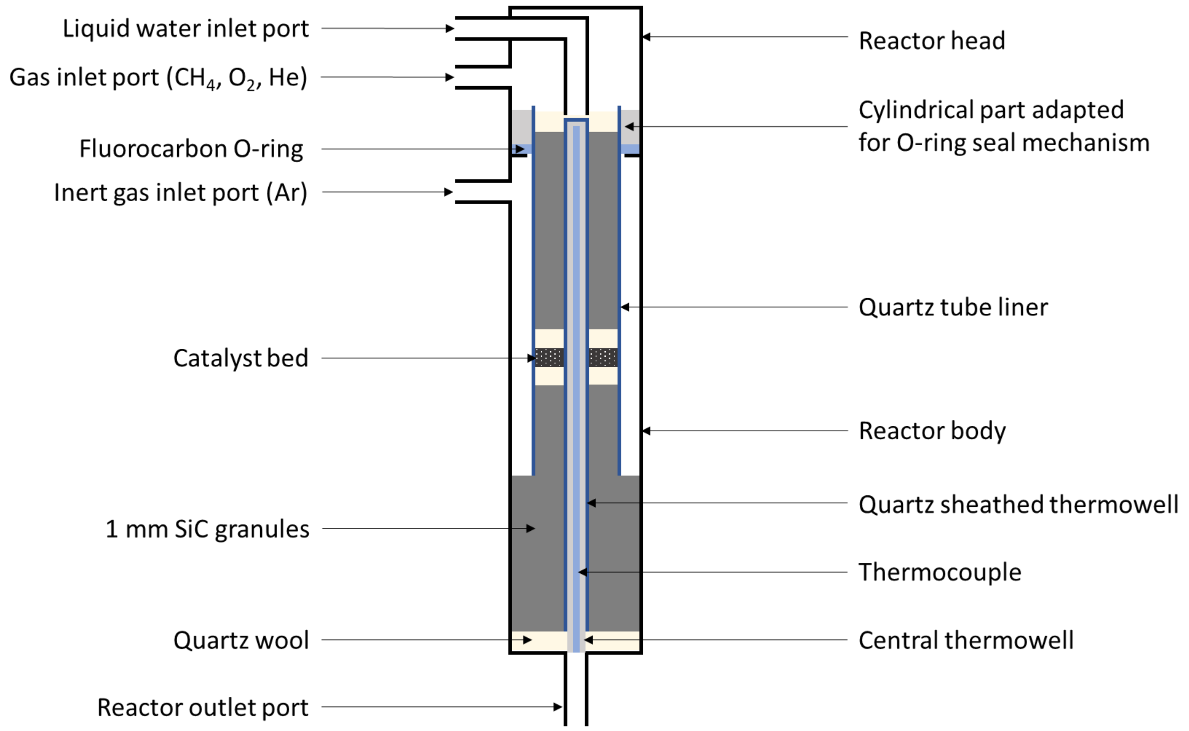


**Figure S.2**: Schematic cross-sectional representation of the trickle bed reactor for the selective oxidation of methane

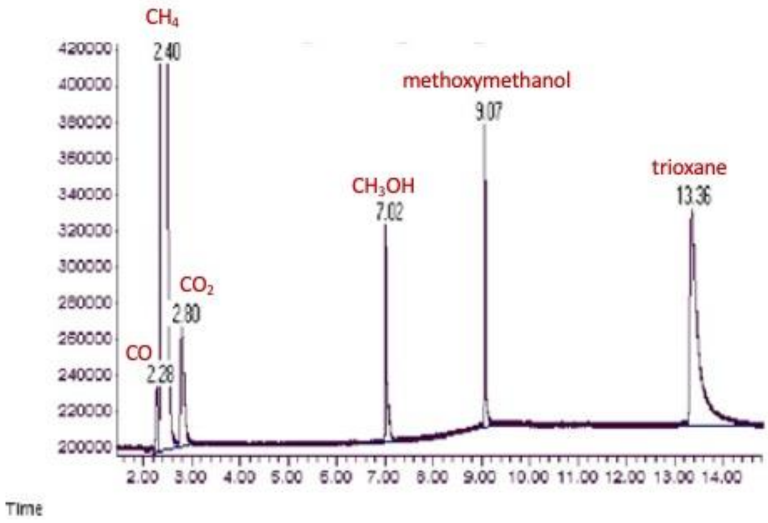


**Figure S.3**: GC-trace obtained during the selective oxidation of methane under the gas-phase conditions

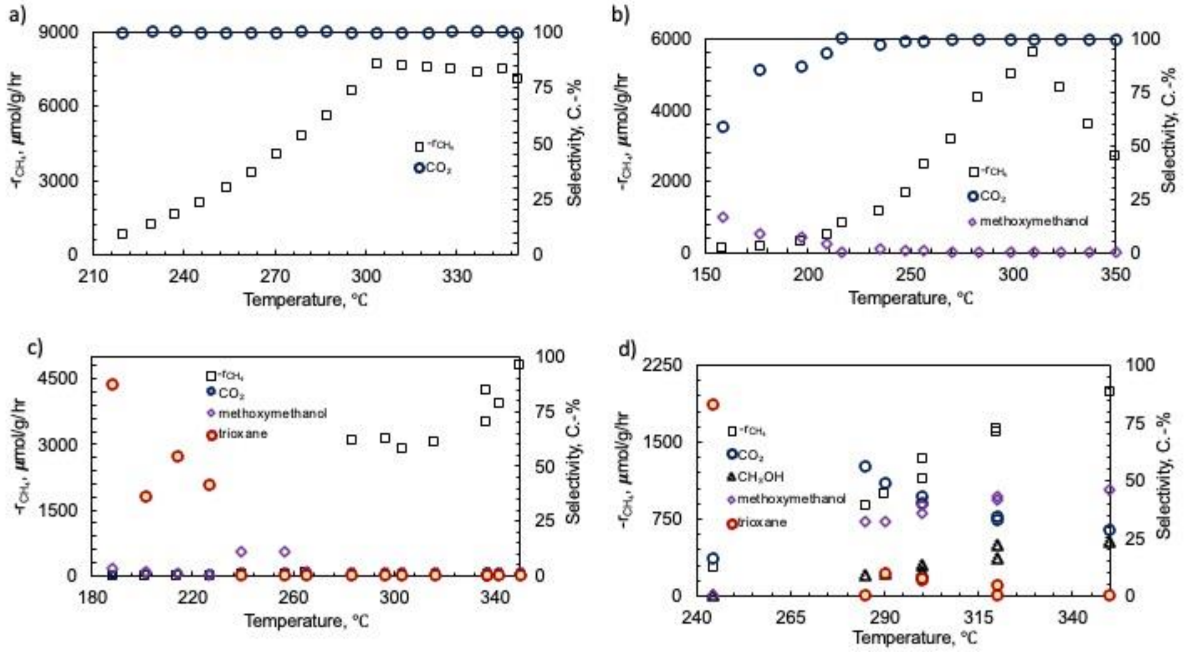


**Figure S.4**: Rate of methane oxidation over 10 wt.% Pt/$TiO_2$ (rutile) conducted in the gas phase reactor, with a $(H_2O/CH_4)_{feed}$ mol/mol ratio of 0 (a), 6 (b), 12 (c) and 50 (d) ($CH_4$=2 $ml_n$/min, $P_{total}$= 20 bar, $O_2$=16 $ml_n$/min. The He flow rate was modified to maintain constant inlet partial pressures of $CH_4$ and $O_2$.

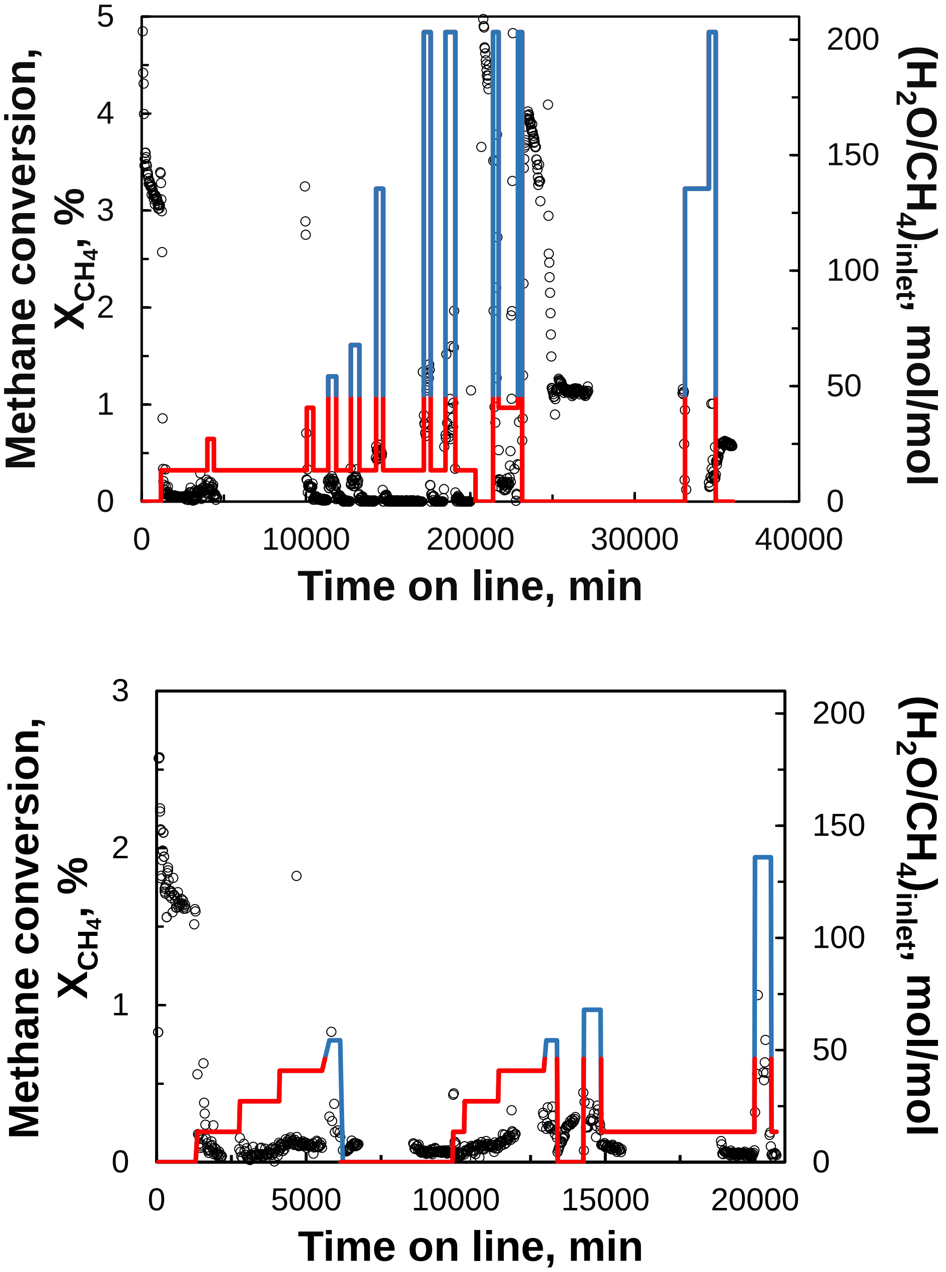


**Figure S.5:** Time-on-stream behavior in the oxidation of methane over 10% Pt/TIO$_2$ (top) and 10% Pt/Al$_2$O$_3$ (bottom) at 220°C, 30 bar and $F_{CH4,0}/W$ = 3.2 mmol/hr/g (inlet partial pressures $p_{CH4}$ = 0.5 bar; $p_{O2}$ = 1.5 bar; red/blue line indicating the water flow rate to the reactor with the blue line indicating flooding conditions in the reactor).

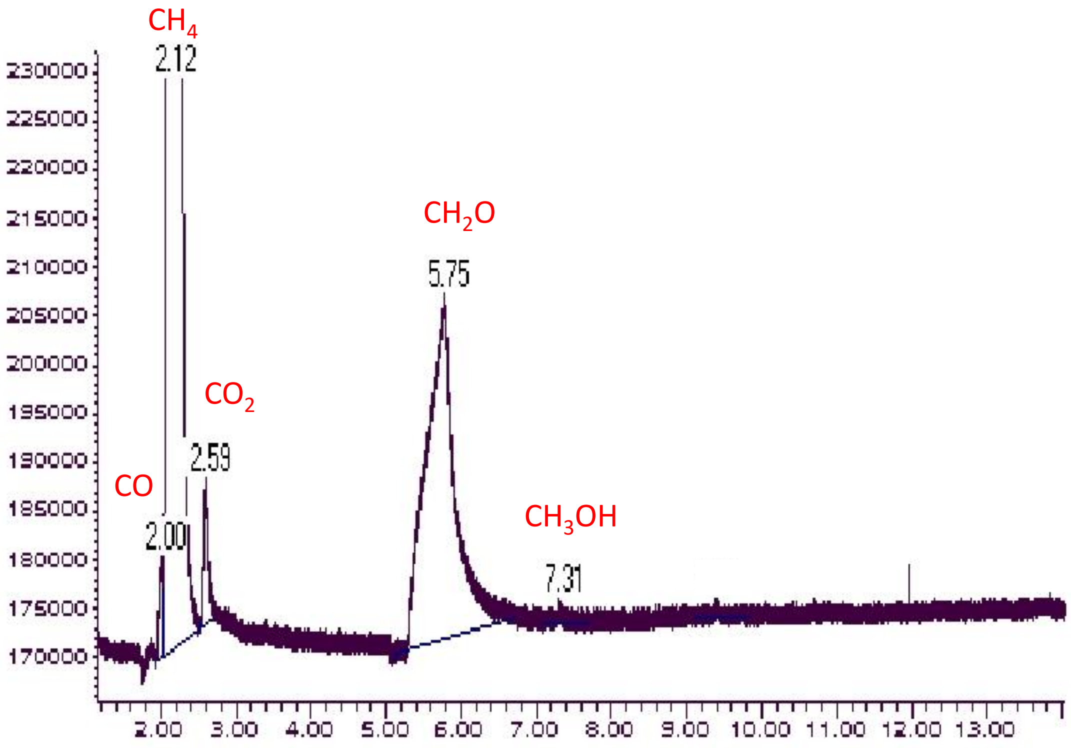


**Figure S6:** GC-trace obtained during the selective oxidation of methane under the trickle-bed conditions over 10% $Pt/Al_2O_3$

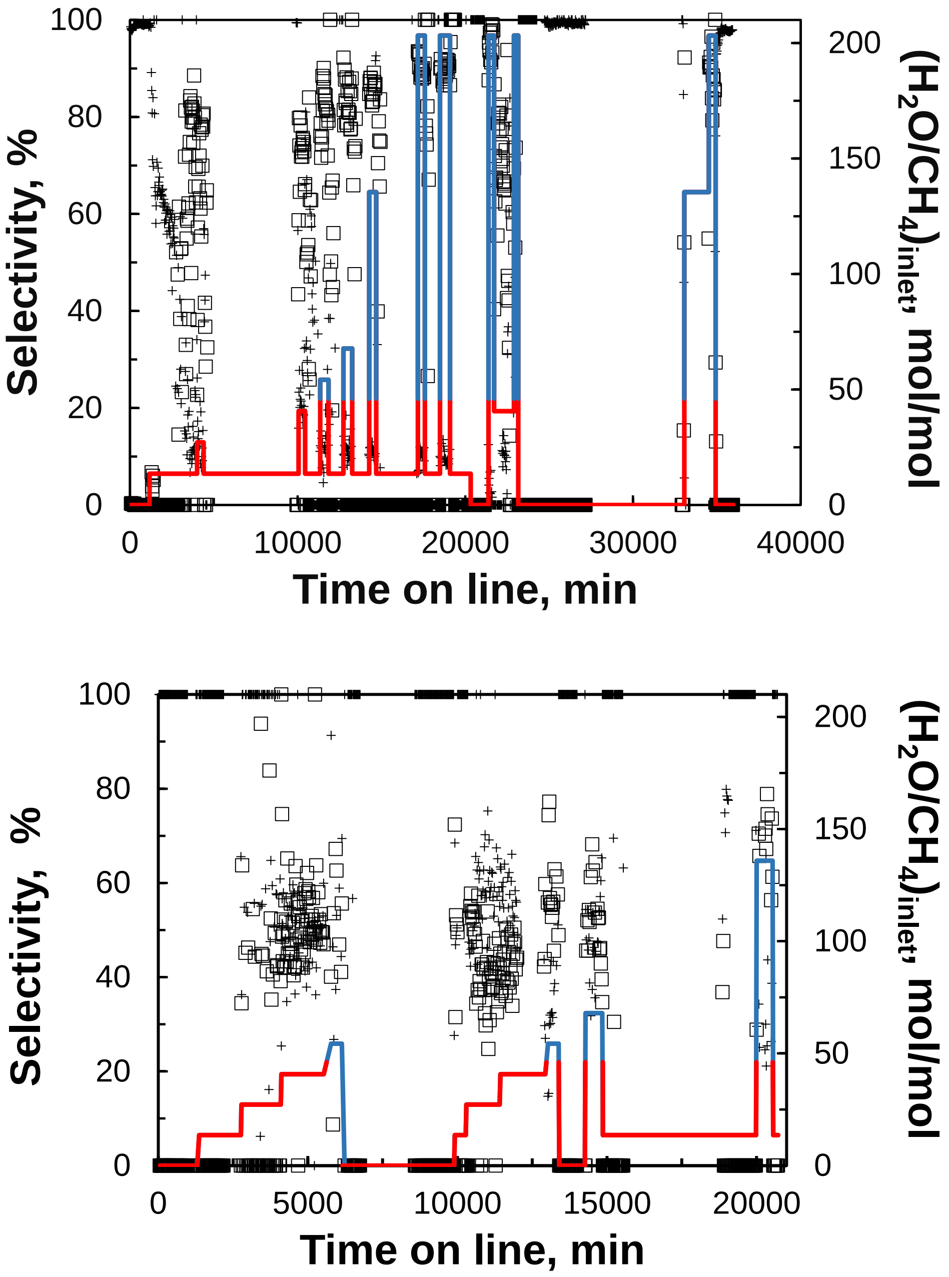


**Figure S.7:** Selectivity for the formation of $CO_2$ (+) and formaldehyde in the oxidation of methane over 10% Pt/$TIO_2$ (top) and 10% Pt/$Al_2O_3$ (bottom) at 220°C, 30 bar and $F_{CH4,0}$/W = 3.2 mmol/hr/g (inlet partial pressures $p_{CH4}$ = 0.5 bar; $p_{O2}$ = 1.5 bar; red/blue line indicating the water flow rate to the reactor with the blue line indicating flooding conditions in the reactor).

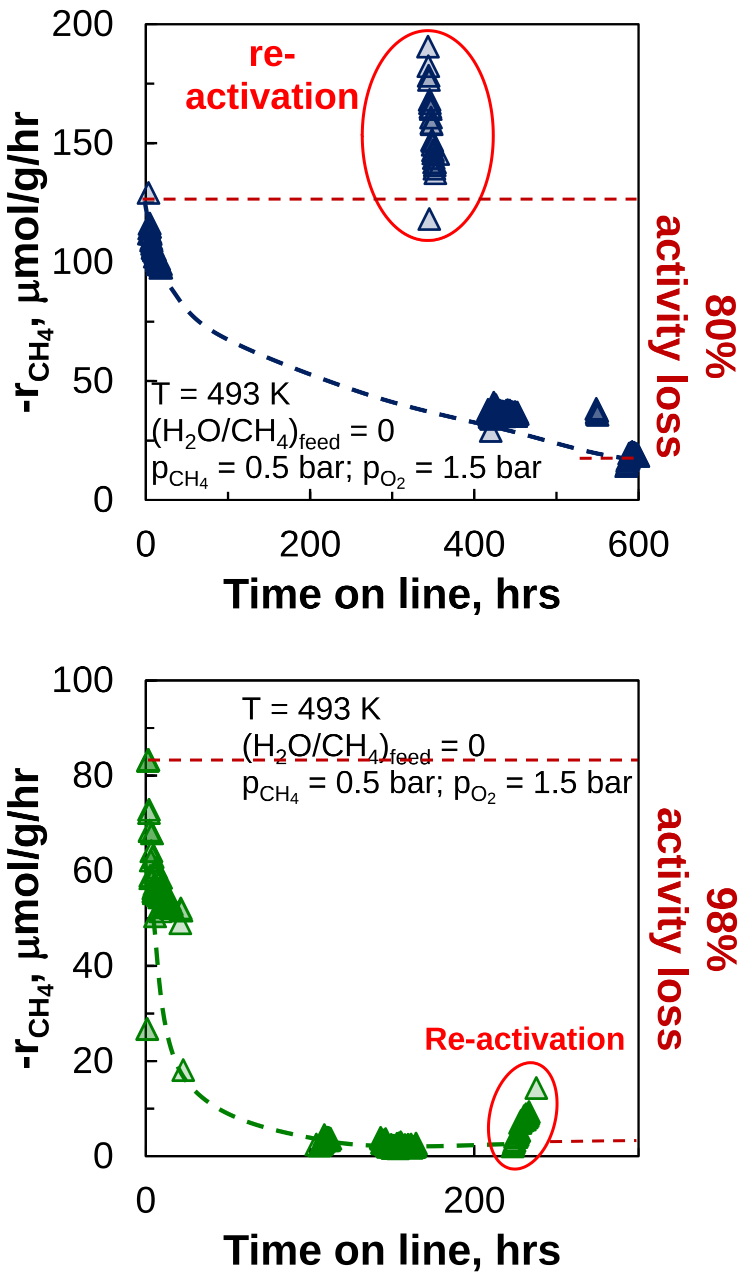


**Figure S8:** Catalyst activity as a function of time on stream over Pt/$TiO_2$ (top) and Pt/Al2O3 (bottom) (220°C, 30 bar and $F_{CH4,0}/W$ = 3.2 mmol/hr/g; inlet partial pressures $p_{CH4}$ = 0.5 bar; $p_{O2}$ = 1.5 bar, $(H_2O/CH_4)_{feed}$ = 0)

## Section II: DFT Studies

Spin polarised quantum chemical calculations were performed using VASP [S2-S4] using the GGA-PBE functional [S5] with dispersion correction according to Grimme et al. with Becke-Jonson damping [S6,S7]. The PAW pseudopotentials were used to describe the electron-ion interaction [S3]. Smearing using 1st order Methfessel-Paxton method [S8] was applied with $\sigma=0.05\ eV$ for surface calculations. Brillouin zone was sampled using a Γ-centred Monkhorst-Pack grid [S9] with a plane wave cut-off energy of 500 eV (grid size (√3x√3): 12 x 12 x1; (2x2): 10 x 10 x 1; (3x3): 7 x 7 x 1, respectively). The lattice parameter for bulk FCC platinum was evaluated to be 3.9246 Å with a bulk modulus of 301 GPa in good agreement with the experimental values of 3.9231 Å and 282.7 GPa, respectively [S10].

A five layer Pt(111) slab with a 15 Å vacuum layer was used in this study. For the geometry optimizations, all the atoms were allowed to relax except the bottom two layers of the slabs. Surface structures were optimized with a maximum force of 0.01 eV/Å applying dipole correction in the direction perpendicular to the surface (SCF < $10^{-5}$ eV). The optimized structures represent local minimums on the potential energy surface as confirmed by a vibrational analysis. The vibrational modes were obtained allowing only the atoms of the adsorbate to move by 0.015 Å.

The obtained energies were referenced to that of the bare surface and the molecules $CH_4$, $O_2$, $H_2$, H, OH, $H_2O$, methanol, methanediol and formaldehyde, which were optimized by placing each molecule in a 15 Å x 16 Å x 17 Å box (plane wave cut-off energy: 1000 eV; Gaussian smearing, σ = 0.005 eV; Gamma-centered k-point grid: 1x1x1; maximum allowable force: 0.01 eV/Å). The obtained bond distances and vibrational modes of the gas phase molecules were compared with experimentally determined values [S10-S12].

The adsorption energy as reported in Tables S3-S5 was determined relative to $H_2O$, $O_2$ and $CH_4$ in the gas phase and normalized with respect to the size of the unit cell taking the (2x2) unit cell as a basis:

$$E_{ads} = \frac{E_{C_\alpha H_\beta O_\gamma\ on\ Pt(111)} - E_{Pt(111)-slab} - \alpha \cdot E_{CH_4} - \frac{\beta-4\cdot\alpha x}{2} \cdot E_{H_2O(g)} - \frac{2\cdot\gamma-\beta+4\cdot\alpha x}{4} \cdot E_{O_2(g)}}{N^{(a\times a)}}$$

and $$\Delta ZPE = \frac{ZPE_{C_\alpha H_\beta O_\gamma\ on\ Pt(111)} - ZPE_{Pt(111)-slab} - \alpha \cdot ZPE_{CH_4} - \frac{\beta-4\cdot\alpha x}{2} \cdot ZPE_{H_2O(g)} - \frac{2\cdot\gamma-\beta+4\cdot\alpha x}{4} \cdot ZPE_{O_2(g)}}{N^{(a\times a)}}$$

with $N^{(\sqrt{3}\times\sqrt{3})} = \frac{3}{4}$; $N^{(2\times2)} = 1$; $N^{(3\times3)} = \frac{9}{4}$.

The most stable structure at a particular condition can be obtained by considering the Gibbs free energy of a particular structure at that condition. The Gibbs free energy associated with a structure was calculated as:

$$G_{structure} = E^{elec}_{structure} - E^{elec}_{bare\ slab} + h^{vib}_{structure} - T \cdot s^{vib}_{structure}$$

with
- $G_{structure}$: the Gibbs free energy associated with structure (slab + adsorbate)
- $E^{elec}_{structure}$: the electronic energy of the structure (slab + adsorbate)
- $E^{elec}_{slab}$: the electronic energy of the bare slab
- $h^{vib}_{stucture}$: the enthalpy correction due to vibration of the adsorbate
- $s^{vib}_{stucture}$: the entropy correction due to vibration of the adsorbate

After determining the Gibbs free energy of structures on the surface, surface phase diagrams of species on Pt(111) were determined in the presence of water and $O_2$ and in the presence of water, $O_2$, and $CH_4$. Numerous configurations can be obtained considering different species, vacant sites, and different adsorption geometries. Hence, only 63 configurations containing O, OH and $H_2O$ were considered for the surface phase diagram on Pt(111) exposed to water and $O_2$ (see Table S.2) A much more limited additional set (10 different configurations) was considered when dealing with methyl and methoxy species on the surface was investigated to construct a surface phase diagram on Pt(111) exposed to water, $O_2$ and $CH_4$ (see Table S.3 and S.4).

The structure with the lowest Gibbs free energy at a given chemical potential of water, oxygen and methane (as determined by DFT) can be found by searching for the minimum value for:

$$G = \min_i \left( \frac{G_{structure,i} - \alpha \cdot \mu_{CH_4(g)} - \frac{\beta - 4 \cdot \alpha x}{2} \cdot \mu_{H_2O(g)} - \frac{2 \cdot \gamma - \beta + 4 \cdot \alpha x}{4} \cdot \mu_{O_2(g)}}{N^{(a \times a)}} \right)$$

with $G_{structure,i}$: the Gibbs free energy associated with structure i (slab + adsorbate)
$\alpha$ the number of C-atoms in the adsorbate
$\beta$ the number of H-atoms in the adsorbate
$\gamma$ the number of O-atoms in the adsorbate
$N^{(a \times a)}$ the number of surface Pt-atoms on one side of the unit cell

The prediction of the chemical potential of oxygen using DFT is improved significantly by relating the enthalpy/chemical potential of oxygen to the DFT-derived enthalpy/chemical potential of hydrogen and water [S13,S14] rather than using the enthalpy/chemical potential of oxygen derived from DFT-generated data:

$$h_{O_2}^{o} = 2 \cdot \left( h_{H_2O}^{o} - h_{H_2}^{o} - \Delta_{f,H_2O} H^0 \right) \qquad \mu_{O_2}^{o} = 2 \cdot \left( \mu_{H_2O}^{o} - \mu_{H_2}^{o} - \Delta_{f,H_2O} G^0 \right)$$

Hence, the derived phase diagrams in this study refer use water and hydrogen as the reference state for gas phase oxygen.

**Table S.2:** Geometric characteristics, energetics, and normal modes of surface structures considered to determine the surface phase diagram of adsorbed O, OH and $H_2O$ on Pt(111)

| | **$\Theta_*$** | **$\Theta_O$** | **$\Theta_{H2O}$** | **$\Theta_{OH}$** | **$E_{ads}$, eV** | **$\Delta_{ads}$ZPE, eV** |
|---|---|---|---|---|---|---|
| 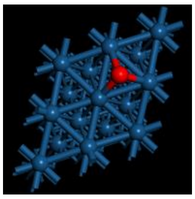 | 0.75 | 0.25 | - | - | -0.974 | 0.026 |
| | $d_{Pt\text{-}O}$: 2.047, 2.047, 2.047 Å<br>(O in fcc hollow on 2x2) | | | | 470, 367, 366 $cm^{-1}$ | |
| 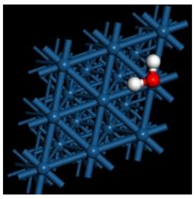 | 0.75 | - | 0.25 | - | -0.464 | 0.073 |
| | $d_{Pt\text{-}O(H,H)}$: 2.410 Å<br>$d_{(Pt)O\text{-}H}$: 0.982, 0.982Å<br>$\angle_{H\text{-}O\text{-}H}$: 104.4°<br>$\angle_{\text{plane through H-O-H and surface}}$: 0.8°<br>($H_2O$ in atop on 2x2) | | | | 3658, 3563, 1551, 566, 508, 165, 151, 91, 84 $cm^{-1}$ | |
| 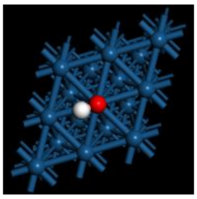 | 0.75 | - | - | 0.25 | -0.594 | 0.028 |
| | $d_{Pt\text{-}O(H)}$: 1.988 Å<br>$d_{(Pt)O\text{-}H}$: 0.980 Å<br>$\angle_{Pt\text{-}O\text{-}H}$: 106.0°<br>(OH in atop on 2x2) | | | | 3650, 928, 518, 126, 118, 80 $cm^{-1}$ | |
| 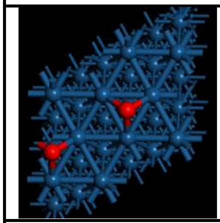 | 0.67 | 0.33 | - | - | -1.049 | 0.011 |
| | $d_{Pt\text{-}O}$: 2.070, 2.071, 2.071 Å<br>(O in fcc hollow on √3x√3) | | | | 448, 260, 259 $cm^{-1}$ | |
| 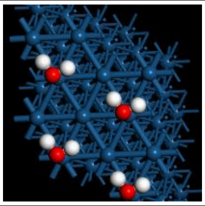 | 0.67 | - | 0.33 | - | -0.630 | 0.081 |
| | $d_{Pt\text{-}O(H,H)}$: 2.447 Å<br>$d_{(Pt)O\text{-}H}$: 0.982, 0.983Å<br>$\angle_{H\text{-}O\text{-}H}$: 103.6°<br>$\angle_{\text{plane through H-O-H and surface}}$: 1.6°<br>($H_2O$ in atop on √3x√3) | | | | 3635, 3539, 1554, 584, 471, 150, 88, 75, 40 $cm^{-1}$ | |
| 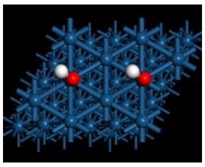 | 0.67 | - | - | 0.33 | -0.848 | 0.039 |
| | $d_{Pt\text{-}O(H)}$: 1.987Å<br>$d_{(Pt)O\text{-}H}$: 0.981 Å<br>$\angle_{Pt\text{-}O\text{-}H}$: 106.4°<br>(OH in atop on √3x√3) | | | | 3638, 940, 520, 152, 107, 81 $cm^{-1}$ | |
| 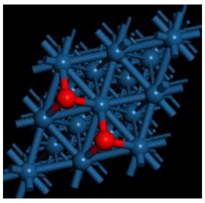 | 0.50 | 0.50 | - | - | -1.899 | 0.062 |
| | $d_{Pt\text{-}O}$: 2.028, 2.028, 2.031, 2.031, 2.033, 2.033 Å<br>(O in fcc hollow on 2x2) | | | | 490, 471, 442, 413, 382, 371 $cm^{-1}$ | |

**Table S.2 (cont.)**

| | $\Theta_*$ | $\Theta_O$ | $\Theta_{H2O}$ | $\Theta_{OH}$ | $E_{ads}$, eV | $\Delta_{ads}$ZPE, eV |
|---|---|---|---|---|---|---|
| 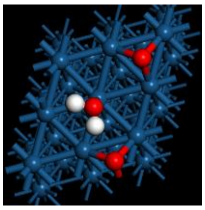 | 0.50 | 0.25 | 0.25 | - | -1.858 | 0.110 |
| | $d_{Pt\text{-}O}$: 2.066, 2.085, 2.090 Å<br>$d_{Pt\text{-}O(H,H)}$: 2.380 Å<br>$d_{(Pt)O\text{-}H}$: 0.980, 0.982 Å<br>$\angle_{H\text{-}O\text{-}H}$: 105.2$^o$<br>$\angle_{\text{plane through H-O-H and surface}}$: 8.3$^o$<br>(O in fcc hollow, $H_2O$ in atop on 2x2) | | | | 3678, 3583, 1561, 624, 591, 437, 333, 311, 213, 183, 109, 74 $cm^{-1}$ | |
| 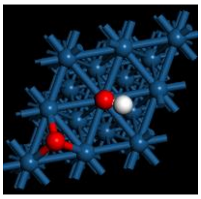 | 0.50 | 0.25 | - | 0.25 | -1.858 | 0.110 |
| | $d_{Pt\text{-}O}$: 2.046, 2.046, 2.059 Å<br>$d_{Pt\text{-}O(H)}$: 1.989 Å<br>$d_{(Pt)O\text{-}H}$: 0.980 Å<br>$\angle_{Pt\text{-}O\text{-}H}$: 107.3$^o$<br>(O in fcc hollow, OH in atop on 2x2) | | | | 3650, 954, 521, 465, 370, 356, 170, 138, 110 $cm^{-1}$ | |
| 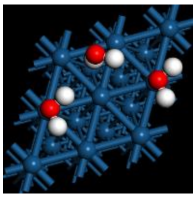 | 0.50 | - | 0.50 | - | -1.335 | 0.167 |
| | $d_{Pt\text{-}O(H,H)}$: 2.397 Å<br>$d_{Pt\text{-}H(O)}$: 2.489, 2.554, 2.711 Å<br>$d_{(Pt)O\text{-}H}$: 0.980, 1.001, 0.979, 0.996 Å<br>$\angle_{H\text{-}O\text{-}H}$: 106.1$^o$, 102.5$^o$<br>$\angle_{\text{plane through H-O-H and surface}}$: 21.6$^o$, 77.6$^o$<br>($H_2O$ in atop and $H_2O$ above hcp hollow on 2x2) | | | | 3674, 3638, 3296, 3192, 1604, 1547, 879, 634, 586, 525, 399, 249, 210, 165, 142, 140, 71, 61 $cm^{-1}$ | |
| 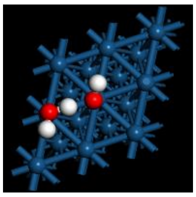 | 0.50 | - | 0.25 | 0.25 | -1.664 | 0.117 |
| | $d_{Pt\text{-}O(H)}$: 2.081 Å<br>$d_{Pt\text{-}O(H,H)}$: 2.186 Å<br>$d_{(Pt)O\text{-}H}$: 0.977 Å<br>$d_{(Pt,H)O\text{-}H}$: 0.977, 1.078 Å<br>$\angle_{Pt\text{-}O\text{-}H}$: 104.9$^o$<br>$\angle_{H\text{-}O\text{-}H}$: 109.1$^o$<br>$\angle_{\text{plane through H-O-H and surface}}$: 20.9$^o$<br>($H_2O$ and OH in atop on 2x2) | | | | 3690, 3690, 1853, 1539, 1269, 872, 738, 620, 422, 386, 307, 229, 164, 135, 109 $cm^{-1}$ | |
| 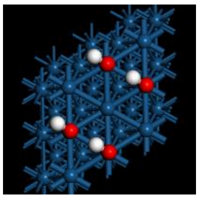 | 0.50 | - | - | 0.50 | -1.639 | 0.103 |
| | $d_{Pt\text{-}O(H)}$: 1.988, 1.988 Å<br>$d_{(Pt)O\text{-}H}$: 0.991, 0.992 Å<br>$\angle_{Pt\text{-}O\text{-}H}$: 106.2$^o$, 106.2$^o$<br>(OH in atop on 2x2) | | | | 3394, 3371, 1144, 1055, 618, 533, 515, 405, 257, 147, 90, 76 $cm^{-1}$ | |
| 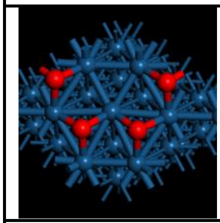 | 0.33 | 0.67 | - | - | -2.047 | 0.082 |
| | $d_{Pt\text{-}O}$: 2.038, 2.038, 2.038, 2.038, 2.038, 2.039 Å<br>(O in fcc hollow on √3x√3) | | | | 503, 430, 428, 420, 388, 386 $cm^{-1}$ | |
| | 0.33 | 0.33 | 0.33 | - | [1] | |
| | | | | | | |

[1] A structure containing $\theta_* = 0.33$, $\theta_O = 0.33$, $\theta_{H2O} = 0.33$ on (√3x√3)-Pt(111) optimises to a structure with $\theta_* = 0.33$, $\theta_{OH} = 0.67$.

**Table S.2 (cont.)**

| | $\Theta_*$ | $\Theta_O$ | $\Theta_{H2O}$ | $\Theta_{OH}$ | $E_{ads}$, eV | $\Delta_{ads}$ZPE, eV |
|---|---|---|---|---|---|---|
| 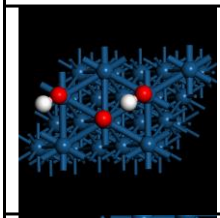 | 0.33 | 0.33 | - | 0.33 | -0.959 | 0.062 |
| | $d_{Pt-O}$: 1.845 Å<br>$d_{Pt-O(H)}$: 1.957 Å<br>$d_{(Pt)O-H}$: 1.003 Å<br>$\angle_{Pt-O-H}$: 105.6° <br>(O and OH atop on √3x√3) | | | | 3139, 1065, 634, 537, 493, 204, 154, 142, 132 $cm^{-1}$ | |
| 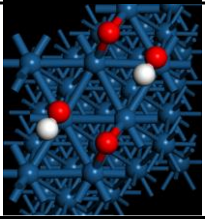 | 0.33 | 0.33 | - | 0.33 | -1.141 | 0.079 |
| | $d_{Pt-O}$: 1.997, 2.004 Å<br>$d_{Pt-O(H)}$: 1.994 Å<br>$d_{(Pt)O-H}$: 0.981 Å<br>$\angle_{Pt-O-H}$: 107.1°<br>(O in bridge and OH atop on √3x√3) | | | | 3639, 906, 500, 476, 367, 317, 186, 168, 150 $cm^{-1}$ | |
| 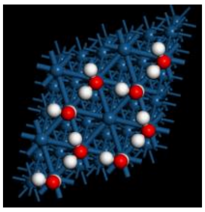 | 0.33 | - | 0.67 | - | -1.994 | 0.246 |
| | $d_{Pt-O(H,H)}$: 2.536 Å<br>$d_{Pt-H(O,H)}$: 2.087 Å<br>$d_{(Pt, H)O-H}$: 0.996, 0.997, 0.993, 1.009 Å<br>$\angle_{H-O-H}$: 106.4°,100.0°<br>$\angle_{plane\ through\ H-O-H\ and\ surface}$: 0.4°, 89.7°<br>($H_2O$ alternating in atop and perpendicular orientation on √3x√3) | | | | 3402, 3272, 3254, 3017, 1640, 1544, 956, 875, 854, 588, 502, 429, 280, 233, 231, 114, 70, 43 $cm^{-1}$ | |
| 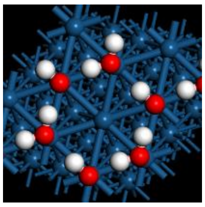 | 0.33 | - | 0.33 | 0.33 | -2.831 | 0.225 |
| | $d_{Pt-O(H)}$: 2.101 Å<br>$d_{Pt-O(H,H)}$: 2.201Å<br>$d_{(Pt)O-H}$: 0.985 Å<br>$d_{(Pt,H)O-H}$:1.017, 1.018 Å<br>$\angle_{Pt-O-H}$: 102.8°<br>$\angle_{H-O-H}$: 112.1°<br>$\angle_{plane\ through\ H-O-H\ and\ surface}$: 14.9 °<br>($H_2O$ and OH in atop on √3x√3) | | | | 3528, 2868, 2823, 1537, 1071, 1016, 961, 958, 623, 408, 336, 254, 197, 140, 128 $cm^{-1}$ | |
| 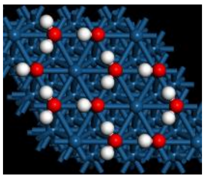 | 0.33 | - | 0.33 | 0.33 | -2.756 | 0.232 |
| | $d_{Pt-O(H)}$: 2.101, 2.101, 2.101 Å<br>$d_{Pt-O(H,H)}$:2.200, 2.200, 2.200 Å<br>$d_{(Pt)O-H}$: 0.985, 0.985, 0.985 Å<br>$d_{(Pt,H)O-H}$:1.017, 1.017, 1.017 Å<br>1.019, 1.019, 1.019 Å<br>$\angle_{Pt-O-H}$: 102.7°, 102.7°, 102.7°<br>$\angle_{H-O-H}$: 112.4°, 112.4°, 112.4°<br>$\angle_{plane\ through\ H-O-H\ and\ surface}$: 14.8°, 14.8°, 14.8°<br>($H_2O$ and OH in atop on 3x3) | | | | 3529, 3524, 3520, 2918, 2916, 2869, 2823, 2814, 2812, 1552, 1552, 1539, 1084, 1084, 1073, 1021, 986, 985, 975, 973, 965,<br>960, 846, 846, 773, 771, 626, 408, 397, 396, 338, 321, 317, 257, 228, 227, 205, 204, 200, 186, 185, 173, 171, 141, 134 $cm^{-1}$ | |
| 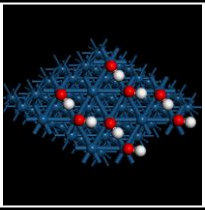 | 0.33 | - | 0.67 | - | -2.270 | 0.152 |
| | $d_{Pt-O-(H)}$: 1.984, 1.984 Å<br>$d_{(Pt)O-H}$: 1.008, 1.009 Å<br>$\angle_{Pt-O-H}$: 104.4°, 104.7°<br>(OH atop on √3x√3) | | | | 3068, 2995, 1201, 1154, 823, 682, 541, 505, 331, 203, 160, 121 $cm^{-1}$ | |
| 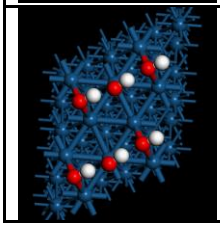 | 0.33 | - | 0.67 | - | -2.155 | 0.137 |
| | $d_{Pt-O-(H)}$: 2.009, 2.119, 2.178 Å<br>$d_{(Pt)O-H}$: 0.995, 1.037 Å<br>$\angle_{Pt-O-H}$: 103.7°, 104.2°, 105.9°<br>(bridging OH + OH atop on √3x√3) | | | | 3252, 2427, 1259, 1077, 1009, 626, 592, 486, 390, 206, 188, 86 $cm^{-1}$ | |

**Table S.2 (cont.)**

| | $\Theta_*$ | $\Theta_O$ | $\Theta_{H2O}$ | $\Theta_{OH}$ | $E_{ads}$, eV | $\Delta_{ads}$ZPE, eV |
|---|---|---|---|---|---|---|
| 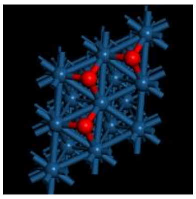 | 0.25 | 0.75 | - | - | -1.700 | 0.091 |
| | $d_{Pt\text{-}O}$: 2.028, 2.028, 2.028, 2.029,<br>2.029, 2.029, 2.034, 2.035<br>2.035 Å<br>(O on fcc hollow on 2x2) | | | | 494, 469, 444, 444, 423, 422,<br>377, 377, 361 $cm^{-1}$ | |
| 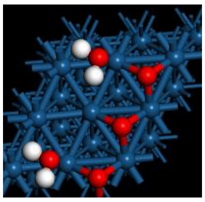 | 0.25 | 0.50 | 0.25 | - | -2.218 | 0.117 |
| | $d_{Pt\text{-}O}$: 2.028, 2.032, 2.033, 2.045,<br>2.051, 2.052Å<br>$d_{Pt\text{-}O(H,H)}$:3.327Å<br>$d_{(H)O\text{-}H}$: 0.973, 0.977 Å<br>$\angle_{H\text{-}O\text{-}H}$: 104.8°<br>$\angle_{\text{plane through H-O-H and surface}}$: 48.4°<br>(O on fcc hollow and $H_2O$ atop on 2x2) | | | | 3780, 3673, 1582, 488, 465, 446,<br>402, 399, 374, 370, 220, 189, 117,<br>56, 27 $cm^{-1}$ | |
| 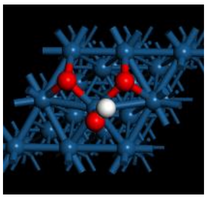 | 0.25 | 0.50 | - | 0.25 | -1.824 | 0.103 |
| | $d_{Pt\text{-}O}$: 2.007, 2.011, 2.046, 2.049,<br>2.061, 2.072 Å<br>$d_{Pt\text{-}O(H)}$: 1.979 Å<br>$d_{(Pt)O\text{-}H}$: 0.982 Å<br>$\angle_{Pt\text{-}O\text{-}H}$: 103.6°<br>(O on fcc hollow and OH atop on 2x2) | | | | 3626, 1036, 527, 494, 467, 449, 416,<br>402, 353, 214, 127, 93 $cm^{-1}$ | |
| 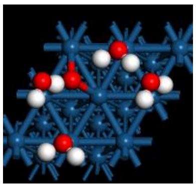 | 0.25 | 0.25 | 0.50 | - | -2.627 | 0.220 |
| | $d_{Pt\text{-}O}$: 2.069, 2.081, 2.121 Å<br>$d_{Pt\text{-}O(H,H)}$:2.295, 3.592 Å<br>$d_{(Pt)O\text{-}H}$: 0.973, 0.982, 0.995, 1.021 Å<br>$\angle_{H\text{-}O\text{-}H}$: 105.2°, 105.1°<br>$\angle_{\text{plane through H-O-H and surface}}$: 28.8°, 47.6°<br>(O on fcc hollow, $H_2O$ atop on 2x2) | | | | 3780, 3586, 3312, 2838, 1629, 1585,<br>1031, 768, 714, 575, 469, 406, 351,<br>343, 322, 280, 211, 169, 140, 105, 48<br>$cm^{-1}$ | |
| | 0.25 | 0.25 | 0.25 | 0.25 | [2] | |
| | | | | | | |
| 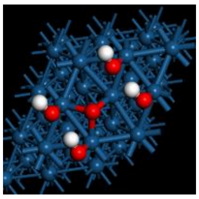 | 0.25 | 0.25 | - | 0.50 | -1.460 | 0.141 |
| | $d_{Pt\text{-}O}$: 1.984, 2.043, 2.066 Å<br>$d_{Pt\text{-}O(H)}$: 1.989, 2.005 Å<br>$d_{(Pt)O\text{-}H}$: 0.990, 0.993 Å<br>$\angle_{Pt\text{-}O\text{-}H}$: 105.4°, 106.2°<br>(O on fcc hollow, OH atop on 2x2) | | | | 3451, 3343, 1166, 1087, 571, 526,<br>517, 496, 423, 400, 374, 273, 194,<br>121, 64 $cm^{-1}$ | |
| 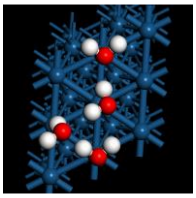 | 0.25 | - | 0.75 | - | -2.024 | 0.289 |
| | $d_{Pt\text{-}O(H,H)}$: 2.314,3.357 Å<br>$d_{Pt\text{-}H(O)}$: 2.311 Å<br>$d_{(Pt)O\text{-}H}$: 0.980, 0.982, 0.987, 0.993,<br>1.000, 1.023 Å<br>$\angle_{H\text{-}O\text{-}H}$: 103.8°, 103.9°, 105.8°<br>$\angle_{\text{plane through H-O-H and surface}}$: 23.8°, 30.8°, 78.1°<br>($H_2O$ on/above atop on 2x2) | | | | 3627, 3611, 3477, 3354, 3222, 2777,<br>1673, 1602, 1574, 1097, 804, 677,<br>646, 625, 545, 515, 454, 389, 365,<br>257, 204, 172, 146, 122, 94, 68, 50<br>$cm^{-1}$ | |

[2] Starting from a structure containing $\theta_* = 0.25$, $\theta_O = 0.25$, $\theta_{H2O} = 0.25$ and $\theta_{OH} = 0.25$ on (2x2)-Pt(111)optimises to a structure with $\theta_* = 0.25$ and $\theta_{OH} = 0.75$.

**Table S.2 (cont.)**

| | $\Theta_*$ | $\Theta_O$ | $\Theta_{H2O}$ | $\Theta_{OH}$ | $E_{ads}$, eV | $\Delta_{ads}ZPE$, eV |
|---|---|---|---|---|---|---|
| 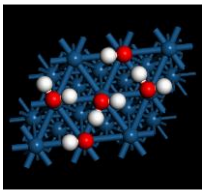 | 0.25 | - | 0.50 | 0.25 | -2.591 | 0.253 |
| | $d_{Pt\text{-}O(H)}$: 2.149 Å<br>$d_{Pt\text{-}O(H,H)}$: 2.323, 2.333 Å<br>$d_{(Pt,H)O\text{-}H}$: 0.983, 1.043 Å<br>0.984, 1.043 Å<br>$d_{(Pt)O\text{-}H}$: 0.975 Å<br>$\angle_{H\text{-}O\text{-}H}$: 107.8°, 107.9°<br>$\angle_{Pt\text{-}O\text{-}H}{}^{a}$: 96.4°<br>$\angle_{\text{plane through H-O-H and surface}}$: 16.6°, 16.6°<br>($H_2O$ and OH atop on 2x2) | | | | 3706, 3558, 3505, 2469, 2305, 1681, 1533, 1171, 1135, 868, 792, 722, 689, 630, 519, 393, 386, 337, 225, 196, 180, 147, 115, 113 $cm^{-1}$ | |
| 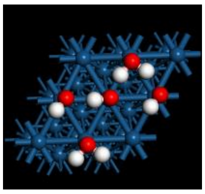 | 0.25 | - | 0.25 | 0.50 | -2.873 | 0.210 |
| | $d_{Pt\text{-}O(H)}$: 2.034,2.066 Å<br>$d_{Pt\text{-}O(H,H)}$: 2.126 Å<br>$d_{(Pt,H)O\text{-}H}$: 1.017, 1.064 Å<br>$d_{(Pt)O\text{-}H}$: 0.978, 1.004 Å<br>$\angle_{H\text{-}O\text{-}H}$: 95.9°<br>$\angle_{Pt\text{-}O\text{-}H}$: 100.1°, 100.1°<br>$\angle_{\text{plane through H-O-H and surface}}$: 9.5°<br>($H_2O$ and OH atop on 2x2) | | | | 3670, 3109, 2942, 2114, 1589, 1272, 1166, 1097, 942, 876, 646, 492, 465, 451, 425, 324, 284, 206, 151, 134, 133 $cm^{-1}$ | |
| 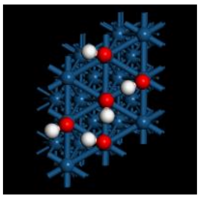 | 0.25 | - | - | 0.75 | -2.365 | 0.165 |
| | $d_{Pt\text{-}O(H)}$: 1.980, 1.988, 1.985 Å<br>$d_{(Pt)O\text{-}H}$: 1.007, 1.007, 1.015 Å<br>$\angle_{Pt\text{-}O\text{-}H}$: 103.4°, 103.7°, 104.6°<br>(OH atop on 2x2) | | | | 3080, 3021, 2865, 1247, 1179, 1135, 933, 697, 605, 541, 519, 492, 358, 270, 245 $cm^{-1}$ | |
| 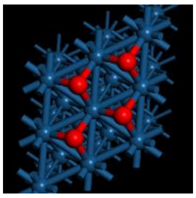 | - | 1.00 | - | - | -0.984 | 0.100 |
| | $d_{Pt\text{-}O}$: 2.041, 2.041, 2.041, 2.044,<br>2.044, 2.044, 2.044, 2.044,<br>2.044, 2.044, 2.044, 2.044 Å<br>(O on fcc-hollow on 2x2) | | | | 496, 429, 428, 427, 419, 417, 416, 369, 368, 366, 310, 305 $cm^{-1}$ | |
| 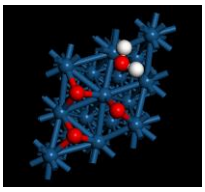 | - | 0.75 | 0.25 | - | -1.818 | 0.171 |
| | $d_{Pt\text{-}O}$: 1.967, 1.968, 1.994, 1.998,<br>2.038, 2.046, 2.052, 2.059 Å<br>$d_{Pt\text{-}O(H,H)}$: 3.265 Å<br>$d_{(H)O\text{-}H}$: 0.973, 0.980 Å<br>$\angle_{H\text{-}O\text{-}H}$: 105.3°<br>$\angle_{\text{plane through H-O-H and surface}}$: 14.8 °<br>(O on fcc-hollow; $H_2O$ above bridge on 2x2) | | | | 3785, 3592, 1579, 577, 546, 510, 487, 455, 441, 425, 411, 379, 298, 272, 217, 118, 94, 79 $cm^{-1}$ | |
| 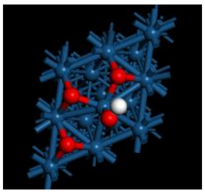 | - | 0.75 | - | 0.25 | -1.517 | 0.136 |
| | $d_{Pt\text{-}O}$: 2.013, 2.015, 2.021, 2.022,<br>2.036, 2.038, 2.044, 2.064,<br>2.072 Å<br>$d_{Pt\text{-}O(H)}$: 1.982 Å<br>$d_{(Pt)\text{-}O\text{-}H}$: 0.982 Å<br>$\angle_{Pt\text{-}O\text{-}H}$: 104.1°<br>(O on fcc-hollow; OH atop on 2x2) | | | | 3632, 1035, 520, 489, 477, 467, 450, 438, 435, 413, 381, 360, 188, 126, 106 $cm^{-1}$ | |

**Table S.2 (cont.)**

| | $\Theta_*$ | $\Theta_O$ | $\Theta_{H2O}$ | $\Theta_{OH}$ | $E_{ads}$, eV | $\Delta_{ads}ZPE$, eV |
|---|---|---|---|---|---|---|
| 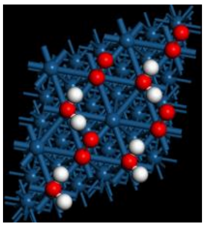 | - | 0.67[3] | 0.33 | - | -1.683[4] | 0.141 |
| | $d_{Pt-O}$: 2.032 Å<br>$d_{O-O}$: 1.344 Å<br>$d_{Pt-O(H,H)}$: 2.074 Å<br>$d_{(H)O-H}$: 0.993, 1.090 Å<br>$\angle_{Pt-O-O}$: 119.7°<br>$\angle_{H-O-H}$: 105.5°<br>$\angle_{plane\ through\ H-O-H\ and\ surface}$: 10.7°<br>($H_2O$ and $O_2$ atop on √3x√3) | | | | 3315, 1647, 1618, 1079, 993, 972, 624, 558, 460, 316, 289, 233, 158, 94, 73 cm$^{-1}$ | |
| 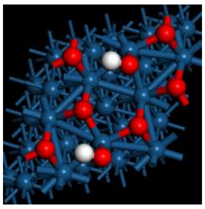 | - | 0.67 | - | 0.33 | -1.830 | 0.161 |
| | $d_{Pt-O}$: 2.015, 2.019, 2.020, 2.027, 2.066, 2.067 Å<br>$d_{Pt-O(H)}$: 1.977 Å<br>$d_{(Pt)O-H}$: 0.983 Å<br>$\angle_{Pt-O-H}$: 104.1°<br>(O in fcc hollow; OH atop on √3x√3) | | | | 3606, 1068, 541, 522, 511, 473, 427, 402, 392, 260, 147, 137 cm$^{-1}$ | |
| 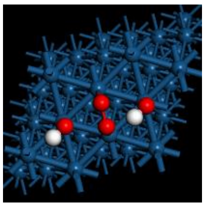 | - | 0.67[3] | - | 0.33 | -1.632 | 0.167 |
| | $d_{Pt-O(O)}$: 2.004, 2.042 Å<br>$d_{O-O}$: 1.389 Å<br>$d_{Pt-O(H)}$: 1.959 Å<br>$d_{(Pt)O-H}$: 0.986 Å<br>$\angle_{Pt-O-O}$: 109.0°, 109.3°<br>$\angle_{Pt-O-H}$: 108.3°<br>($O_2$ on bridge; OH atop on √3x√3) | | | | 3437, 1017, 801, 582, 562, 522, 417, 380, 286, 243, 165, 151 cm$^{-1}$ | |
| 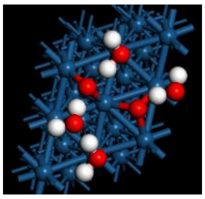 | - | 0.50 | 0.50 | - | -3.174 | 0.247 |
| | $d_{Pt-O}$: 2.032, 2.034, 2.045, 2.047, 2.050 Å<br>$d_{Pt-O(H,H)}$: 2.127, 3.561 Å<br>$d_{(H)O-H}$: 0.976, 1.006, 1.039, 1.043 Å<br>$\angle_{H-O-H}$: 103.3°, 104.5°<br>$\angle_{plane\ through\ H-O-H\ and\ surface}$: 28.2°, 40.7°<br>(O in fcc hollow and bridge; $H_2O$ atop on 2x2) | | | | 3727, 3094, 2510, 2419, 1645, 1610, 1178, 1126, 930, 817, 550, 517, 464, 441, 440, 413, 376, 367, 363, 292, 224, 150, 125, 96 cm$^{-1}$ | |
| 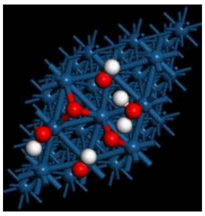 | - | 0.50 | 0.25 | 0.25 | -2.306 | 0.216 |
| | $d_{Pt-O}$: 1.966, 1.975, 2.022, 2.037, 2.050 Å<br>$d_{Pt-O(H)}$: 2.031 Å<br>$d_{Pt-O(H,H)}$: 2.166 Å<br>$d_{(Pt)O-H}$: 0.995 Å<br>$d_{(H)O-H}$: 1.032, 1.043 Å<br>$\angle_{Pt-O-H}$: 98.1°<br>$\angle_{H-O-H}$: 102.8°<br>$\angle_{plane\ through\ H-O-H\ and\ surface}$: 0.0°<br>(O in fcc hollow and bridge; $H_2O$ and OH atop on 2x2) | | | | 3324, 2604, 2374, 1613, 1192, 1099, 1020, 861, 617, 560, 530, 507, 477, 444, 413, 362, 343, 302, 240, 169, 138 cm$^{-1}$ | |
| 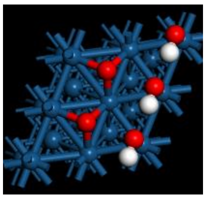 | - | 0.50 | - | 0.50 | -1.942 | 0.184 |
| | $d_{Pt-O}$: 2.012, 2.012, 2.017, 2.017, 2.022, 2.022 Å<br>$d_{Pt-O(H)}$: 2.010, 2.011 Å<br>$d_{(Pt)O-H}$: 0.992, 0.992 Å<br>$\angle_{Pt-O-H}$: 105.0°, 105.1°<br>(O in fcc hollow; OH atop on 2x2) | | | | 3405, 3380, 1152, 1066, 560, 512, 502, 501, 473, 461, 456, 434, 404, 388, 256, 200, 195, 131 cm$^{-1}$ | |

[3] In this configuration molecularly adsorbed $O_2$ is on the surface rather than 2O

[4] The same coverage with O in the fcc hollow site is more stable (Eads = -2.527 eV), but vibrational analysis resulted in a single imaginary frequency

**Table S.2 (cont.)**

| | Θ* | ΘO | ΘH2O | ΘOH | Eads, eV | ΔadsZPE, eV |
|---|---|---|---|---|---|---|
| | - | 0.33 | 0.67 | | [5] | |
| | | | | | | |
| 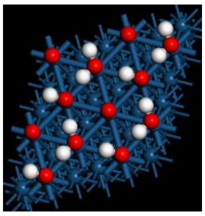 | - | 0.33 | 0.33 | 0.33 | -3.064 | 0.262 |
| | $d_{Pt\text{-}O}$: 1.840 Å<br>$d_{Pt\text{-}O(H)}$: 2.053 Å<br>$d_{Pt\text{-}O(H,H)}$: 2.142 Å<br>$d_{(Pt)O\text{-}H}$: 0.987 Å<br>$d_{(H)O\text{-}H}$: 1.016, 1.017 Å<br>$\angle_{Pt\text{-}O\text{-}H}$: 104.0°<br>$\angle_{H\text{-}O\text{-}H}$: 110.7°<br>$\angle_{\text{plane through H-O-H and surface}}$: 17.9°<br>(O, $H_2O$ and OH atop on √3x√3) | | | | 3491, 2903, 2874, 1494, 1104, 1073, 1024, 830, 656, 502, 447, 375, 294, 278, 238,<br>205, 156, 143 $cm^{-1}$ | |
| 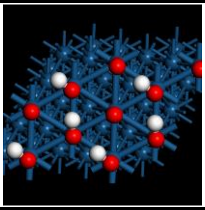 | - | 0.33 | - | 0.67 | -2.259 | 0.173 |
| | $d_{Pt\text{-}O}$: 1.831 Å<br>$d_{Pt\text{-}O(H)}$: 1.971, 1.971 Å<br>$d_{(Pt)O\text{-}H}$: 1.005, 1.005 Å<br>$\angle_{Pt\text{-}O\text{-}H}$: 105.4°, 105.4°<br>(O and OH atop on √3x√3) | | | | 3126, 3087, 1180, 1127, 739, 651, 563, 531, 512, 290, 244, 213, 212, 180, 170 $cm^{-1}$ | |
| 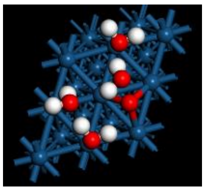 | - | 0.25 | 0.75 | - | -3.313 | 0.321 |
| | $d_{Pt\text{-}O}$: 2.058, 2.063, 2.083 Å<br>$d_{Pt\text{-}O(H,H)}$: 2.315, 3.399,4.371 Å<br>$d_{(H)O\text{-}H}$: 0.975, 0.982, 0.985, 0.988, 1.011, 1.042 Å<br>$\angle_{H\text{-}O\text{-}H}$: 103.3°, 105.4°,105.0°<br>$\angle_{\text{plane through H-O-H and surface}}$: 23.0°, 60.7°, 45.2°<br>(O in fcc hollow; $H_2O$ atop on 2x2) | | | | 3730, 3598, 3525, 3448, 3033, 2467, 1656, 1619, 1583, 1201, 931, 732, 722, 624, 550, 468, 456, 442, 416, 377, 357, 335, 271, 237, 162, 143, 135, 103, 73, 45 $cm^{-1}$ | |
| 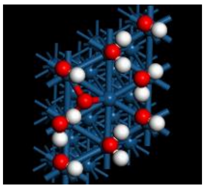 | - | 0.25 | 0.50 | 0.25 | -3.194 | 0.281 |
| | $d_{Pt\text{-}O}$: 2.025, 2.046, 2.082 Å<br>$d_{Pt\text{-}O(H)}$: 2.128Å<br>$d_{Pt\text{-}O(H,H)}$: 2.138, 3.465 Å<br>$d_{(Pt)O\text{-}H}$: 0.978 Å<br>$d_{(H)O\text{-}H}$: 0.978, 1.018, 1.023, 1.052 Å<br>$\angle_{Pt\text{-}O\text{-}H}$: 100.9°<br>$\angle_{H\text{-}O\text{-}H}$: 97.4°, 102.4°<br>$\angle_{\text{plane through H-O-H and surface}}$: 26.9°, 35.4°<br>(O in fcc hollow; $H_2O$, OH atop on 2x2) | | | | 3679, 3670, 2886, 2780, 2332, 1657, 1641, 1210, 1122, 1014, 861, 810, 615, 522, 500, 462, 442, 398, 372, 323, 303, 266, 255, 168, 123, 119, 86 $cm^{-1}$ | |
| 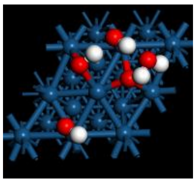 | - | 0.25 | 0.25 | 0.50 | -2.373 | 0.216 |
| | $d_{Pt\text{-}O}$: 1.990, 2.006, 2.073 Å<br>$d_{Pt\text{-}O(H)}$: 2.068, 2.094, 2.159 Å<br>$d_{(Pt)O\text{-}H}$: 1.015, 1.085 Å<br>$d_{(H)O\text{-}H}$: 0.973, 0.995 Å<br>$\angle_{Pt\text{-}O\text{-}H}$: 100.5°, 104.8°, 105.9°<br>$\angle_{H\text{-}O\text{-}H}$: 104.3°<br>$\angle_{\text{plane through H-O-H and surface}}$: 45.9°<br>(O in fcc hollow; $H_2O$ atop, OH bridge on 2x2) | | | | 3772, 3316, 2945, 1725, 1606, 1343, 1306, 1188, 930, 761, 515, 486, 469, 450, 428, 375, 364, 311, 284, 265, 170, 150, 136, 72 $cm^{-1}$ | |

[5] A structure containing $\theta_O = 0.33$, $\theta_{H2O} = 0.67$ on (√3x√3)-Pt(111) optimises to a structure with $\theta_{H2O} = 0.33$, $\theta_{OH} = 0.67$

**Table S.2 (cont.)**

<table>
<tr><th></th><th>$\Theta_*$</th><th>$\Theta_O$</th><th>$\Theta_{H2O}$</th><th>$\Theta_{OH}$</th><th>$E_{ads}$, eV</th><th>$\Delta_{ads}$ZPE, eV</th></tr>
<tr><td rowspan="2">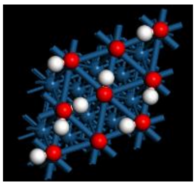</td><td>-</td><td>0.25</td><td>0.25</td><td>0.50</td><td>-3.148</td><td>0.217</td></tr>
<tr><td colspan="4">$d_{Pt\text{-}O}$: 1.861Å<br>$d_{Pt\text{-}O(H)}$: 2.005, 2.006 Å<br>$d_{Pt\text{-}O(H,H)}$: 2.081 Å<br>$d_{(Pt)O\text{-}H}$: 0.994, 0.995 Å<br>$d_{(H)O\text{-}H}$: 1.030, 1.031 Å<br>$\angle_{Pt\text{-}O\text{-}H}$: 104.1°, 104.2°<br>$\angle_{H\text{-}O\text{-}H}$: 113.9°<br>$\angle_{\text{plane through H-O-H and surface}}$: 18.8°<br>(O, $H_2O$ and OH atop on 2x2)</td><td colspan="2">3322, 3312, 2620, 2608, 1404, 1206, 1146, 1075, 1064, 810, 630, 564, 484, 479, 421, 414, 315, 291, 260, 245, 211, 193, 156, 152 $cm^{-1}$</td></tr>
<tr><td rowspan="2">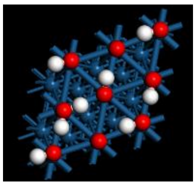</td><td>-</td><td>0.25</td><td>0.25</td><td>0.50</td><td>-3.179</td><td>0.234</td></tr>
<tr><td colspan="4">$d_{Pt\text{-}O}$: 1.880 Å<br>$d_{Pt\text{-}O(H)}$: 1.990, 1.991 Å<br>$d_{Pt\text{-}O(H,H)}$: 2.098 Å<br>$d_{(Pt)O\text{-}H}$: 1.000, 1.005 Å<br>$d_{(H)O\text{-}H}$: 1.018, 1.020 Å<br>$\angle_{Pt\text{-}O\text{-}H}$: 103.9°, 104.3°<br>$\angle_{H\text{-}O\text{-}H}$: 111.1°<br>$\angle_{\text{plane through H-O-H and surface}}$: 17.2°<br>(O, $H_2O$ and OH atop on 2x2)</td><td colspan="2">3198, 3098, 2842, 2810, 1418, 1175, 1141, 1124, 1065, 758, 686, 595, 526, 512, 495, 405, 325, 290, 250, 244, 226, 175, 156, 144 $cm^{-1}$</td></tr>
<tr><td rowspan="2">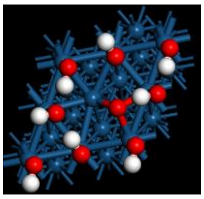</td><td>-</td><td>0.25</td><td>-</td><td>0.75</td><td>-1.971</td><td>0.216</td></tr>
<tr><td colspan="4">$d_{Pt\text{-}O}$: 1.942, 2.043, 2.066 Å<br>$d_{Pt\text{-}O(H)}$: 1.954, 1.973, 2.074 Å<br>$d_{(Pt)O\text{-}H}$: 0.982, 1.010, 1.013 Å<br>$\angle_{Pt\text{-}O\text{-}H}$: 101.2°, 103.6°, 105.3°<br>(O on fcc hollow, OH atop on 2x2)</td><td colspan="2">3618, 3015, 2897, 1241, 1191, 988, 816, 749, 592, 534, 525, 433, 424, 403, 393, 348, 298, 212, 198, 166, 142 $cm^{-1}$</td></tr>
<tr><td rowspan="2">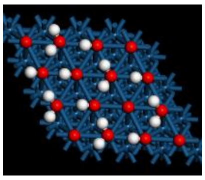</td><td>-</td><td>0.22</td><td>0.33</td><td>0.44</td><td>-3.273</td><td>0.283</td></tr>
<tr><td colspan="4">$d_{Pt\text{-}O}$: 1.843, 1.843 Å<br>$d_{Pt\text{-}O(H)}$: 1.989, 2.052, 2.062, 2.062 Å<br>$d_{Pt\text{-}O(H,H)}$: 2.134, 2.145, 2.148 Å<br>$d_{(Pt)O\text{-}H}$: 0.985, 0.986, 0.986, 0.986 Å<br>$d_{(Pt,H)O\text{-}H)}$:0.999, 1.000, 1.018, 1.018, 1.040, 1.042 Å<br>$\angle_{Pt\text{-}O\text{-}H}$: 104.0°, 104.1°, 104.8°, 106.0°<br>$\angle_{H\text{-}O\text{-}H}$: 103.4°, 104.8°, 108.0°<br>$\angle_{\text{plane through H-O-H and surface}}$: 15.3°, 15.4°, 18.5°<br>(O, $H_2O$, OH atop on 3x3)</td><td colspan="2">3525, 3523, 3495, 3458, 3248, 3227, 2894, 2870, 2455, 2429, 1547, 1542, 1500, 1174, 1169,<br>1132, 1102, 1065, 1041, 1036, 1034, 986, 981, 818, 742, 675, 668, 660, 602, 595, 506, 497, 463, 386, 378, 359, 316, 300, 296, 254, 249, 246, 230, 223, 210, 187, 185, 167, 148, 145, 100 $cm^{-1}$</td></tr>
<tr><td rowspan="2"></td><td>-</td><td>0.11</td><td>0.44</td><td>0.44</td><td>[6]</td><td></td></tr>
<tr><td colspan="4"></td><td colspan="2"></td></tr>
</table>

[6] A structure containing $\theta_O = 0.11$, $\theta_{H2O} = 0.44$ and $\theta_{OH} = 0.44$ on (3x3)-Pt(111) optimises to a structure with $\theta_{H2O} = 0.33$, $\theta_{OH} = 0.67$

**Table S.2 (cont.)**

<table>
<tr><th></th><th>$\Theta_*$</th><th>$\Theta_O$</th><th>$\Theta_{H2O}$</th><th>$\Theta_{OH}$</th><th>$E_{ads}$, eV</th><th>$\Delta_{ads}$ZPE, eV</th></tr>
<tr><td rowspan="2">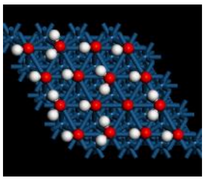</td><td>-</td><td>0.11</td><td>0.33</td><td>0.55</td><td>-3.519</td><td>0.307</td></tr>
<tr><td colspan="4">$d_{Pt-O}$: 1.841 Å<br>$d_{Pt-O(H)}$: 1.998, 2.001, 2.060, 2.064, 2.072 Å<br>$d_{Pt-O(H,H)}$: 2.135, 2.148, 2.151 Å<br>$d_{(Pt)O-H}$: 0.983, 0.983, 0.984, 0.984, 0.986 Å<br>$d_{(Pt,H)O-H}$:0.996, 0.996, 1.022, 1.027, 1.043, 1.046 Å<br>$\angle_{Pt-O-H}$: 104.5°, 104.7°, 104.8, 105.9°, 106.3°<br>$\angle_{H-O-H}$: 107.0°, 107.2°, 110.7°<br>$\angle_{\text{plane through H-O-H and surface}}$: 13.8°, 14.6°, 15.2°<br>(O, $H_2O$, OH atop on 3x3)</td><td colspan="2">3564, 2548, 3512, 2496, 2486, 3328, 3319, 2778, 2688, 2473, 2330, 1572, 1567, 1554, 1203, 1171, 1154, 1124, 1077, 1057, 1032, 1024, 1011, 1001, 988, 877, 720, 685, 651, 607, 555, 543, 506, 486, 473, 450, 433, 421, 414, 386, 380, 348, 332, 332, 317, 295, 293, 288, 277, 260, 256, 235, 228, 215, 203, 179, 151, 147, 144, 110 $cm^{-1}$</td></tr>
<tr><td rowspan="2">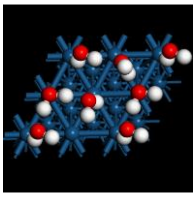</td><td>-</td><td>-</td><td>1.00</td><td>-</td><td>-2.733</td><td>0.389</td></tr>
<tr><td colspan="4">$d_{Pt-O(H,H)}$ : 2.240 Å<br>$d_{Pt-H(O)}$: 2.370, 2.377, 4.946 Å<br>$d_{(Pt)O-H}$: 1.036, 1.036 Å<br>$d_{(Pt)O-H}$: 0.972,0.988, 0.989, 0.990, 0.998, 1.007 Å Å<br>$\angle_{H-O-H}$: 100.7°, 104.8°, 104.8°, 105.8°<br>$\angle_{\text{plane through H-O-H and surface}}$: 26.3°, 60.7°, 61.4°, 89.8°<br>($H_2O$, OH on/above atop on 2x2)</td><td colspan="2">3795, 3423, 3392, 3312, 3231, 3051, 2607, 2484, 1676, 1633, 1589, 1585, 1200, 1143, 1033, 847, 843, 739, 594, 560, 526, 470, 465, 398, 351, 338, 314, 245, 235, 171, 161, 155, 129, 109, 59, 49 $cm^{-1}$</td></tr>
<tr><td rowspan="2">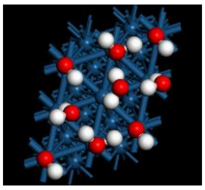</td><td>-</td><td>-</td><td>0.75</td><td>0.25</td><td>-3.118</td><td>0.312</td></tr>
<tr><td colspan="4">$d_{Pt-O(H)}$: 2.071 Å<br>$d_{Pt-O(H,H)}$: 2.145, 3.222, 4.268 Å<br>$d_{(Pt)O-H}$: 0.981 Å<br>$d_{(H)O-H}$: 0.974, 0.991, 0.999, 1.014, 1.025, 1.094 Å<br>$\angle_{Pt-O-H}$: 108.3°<br>$\angle_{H-O-H}$: 106.6°, 102.0°,105.7°<br>$\angle_{\text{plane through H-O-H and surface}}$: 26.1°, 81.4°, 52.8°<br>($H_2O$, OH on/above atop on 2x2)</td><td colspan="2">3757, 3594, 3390, 3213, 3003, 2706, 1635, 1618, 1588, 1576, 1336, 1245, 1072, 929, 897, 736, 719, 554, 483, 469, 427, 396, 355, 327, 300, 251, 213, 169, 155, 139, 101, 78, 52 $cm^{-1}$</td></tr>
<tr><td rowspan="2">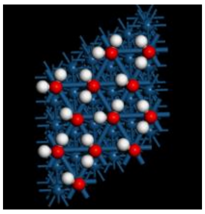</td><td>-</td><td>-</td><td>0.67</td><td>0.33</td><td>-3.241</td><td>0.398</td></tr>
<tr><td colspan="4">$d_{Pt-O(H)}$: 2.186 Å<br>$d_{Pt-O(H,H)}$: 2.340, 2.362 Å<br>$d_{(Pt)O-H}$: 0.983 Å<br>$d_{(H)O-H}$: 0.991, 0.992, 0.996, 0.997 Å<br>$\angle_{Pt-O-H}$: 105.2°<br>$\angle_{H-O-H}$: 106.4°, 106.3°<br>$\angle_{\text{plane through H-O-H and surface}}$: 10.6°, 11.1° ($H_2O$, OH on atop on √3x√3)</td><td colspan="2">3569, 3370, 3332, 3241, 3186, 1639, 1611, 922, 897, 823, 796, 748, 733, 641, 520, 354, 338, 317, 288, 280, 191, 115, 110, 84 $cm^{-1}$</td></tr>
<tr><td rowspan="2">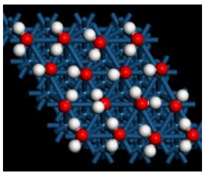</td><td>-</td><td>-</td><td>0.55</td><td>0.44</td><td>-3.604</td><td>0.332</td></tr>
<tr><td colspan="4">$d_{Pt-O(H)}$: 2.100, 2.102, 2.121, 2.138 Å<br>$d_{Pt-O(H,H)}$: 2.153, 2.161, 2.183, 2.211, 3.386 Å<br>$d_{(Pt)O-H}$: 0.972, 0.981, 0.982, 0.996 Å<br>$d_{(H)O-H}$: 0.972, 0.990, 0.991, 0.998, 1.004, 1.010, 1.024, 1.029, 1.034, 1.042 Å<br>$\angle_{Pt-O-H}$: 100.6°, 101.0°, 105.3°, 107.2°<br>$\angle_{H-O-H}$: 102.9°, 105.5°, 110.0°, 110.4 °, 111.0°<br>$\angle_{\text{plane through H-O-H and surface}}$: 6.3°, 12.9°, 14.5°, 60.8°<br>($H_2O$, OH on atop on 3x3)</td><td colspan="2">3739, 3716, 3595, 3494, 3388, 3312, 3277, 3179, 3162, 2886, 2772, 2585, 2478, 2456, 1626, 1612, 1564, 1537, 1511, 1188, 1164, 1154, 1098, 1065, 1057, 1026, 1016, 1003, 956, 929, 922, 866, 857, 820, 804, 705, 689, 640, 569, 456, 423, 413, 402, 388, 384, 372, 348, 339, 328, 307, 299, 287, 277, 272, 259, 253, 250, 239, 221, 217, 205, 191, 177, 140, 135, 127, 111, 88, 40 $cm^{-1}$</td></tr>
</table>

**Table S.2 (cont.)**

<table>
<tr><th></th><th>$\Theta_*$</th><th>$\Theta_O$</th><th>$\Theta_{H2O}$</th><th>$\Theta_{OH}$</th><th>$E_{ads}$, eV</th><th>$\Delta_{ads}$ZPE, eV</th></tr>
<tr><td rowspan="2">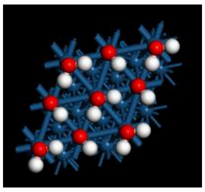</td><td>-</td><td>-</td><td>0.50</td><td>0.50</td><td>-4.039</td><td>0.383</td></tr>
<tr><td colspan="4">$d_{Pt-O(H)}$: 2.123, 2.124 Å<br>$d_{Pt-O(H,H)}$: 2.173, 2.174 Å<br>$d_{(Pt)O-H}$: 0.983, 0.983 Å<br>$d_{(H)O-H}$: 1.011, 1.012, 1.012, 1.013 Å<br>$\angle_{Pt-O-H}$: 105.0°, 104.9°<br>$\angle_{H-O-H}$: 109.7°, 109. 7°<br>$\angle_{\text{plane through H-O-H and surface}}$: 14.0°, 14.1° ($H_2O$, OH on atop on 2x2)</td><td colspan="2">3553, 3502, 2999, 2978, 2909, 2892, 1566, 1537, 1086, 1073, 1069, 1025, 947, 893, 816, 796, 725, 687, 420, 394, 383, 340, 331, 300, 282, 269, 222, 176, 139, 137 $cm^{-1}$</td></tr>
<tr><td rowspan="2">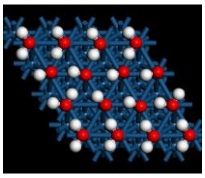</td><td>-</td><td>-</td><td>0.44</td><td>0.55</td><td>-3.846</td><td>0.357</td></tr>
<tr><td colspan="4">$d_{Pt-O(H)}$: 2.060, 2.069, 2.072, 2.077, 2.121 Å<br>$d_{Pt-O(H,H)}$: 2.144, 2.145, 2.157, 2.237 Å<br>$d_{(Pt)O-H}$: 0.976, 0.981, 0.989, 0.990, 0.991 Å<br>$d_{(H)O-H}$: 0.989, 0.993, 1.009, 1.014, 1.023, 1.031, 1.031, 1.043 Å<br>$\angle_{Pt-O-H}$: 102.5°, 103.3°, 104.2°, 105.2°, 106.6°<br>$\angle_{H-O-H}$: 108.2°, 108.8°, 109.4°, 109.5°<br>$\angle_{\text{plane through H-O-H and surface}}$: 14.2°, 14.2°, 15.8°, 17.4°<br>($H_2O$, OH on atop on 3x3)</td><td colspan="2">3662, 3560, 3427, 3406, 3394, 3356, 3322, 3005, 2899, 2771, 2594, 2589, 2396, 1586, 1574, 1543, 1513, 1193, 1171, 1141, 1120, 1090, 1063, 1049, 1027, 1006, 1000, 983, 950, 925, 862, 845, 756, 743, 714, 665, 647, 571, 453, 447, 440, 426, 407, 399, 393, 379, 370, 366, 351, 332, 324, 306, 300, 291, 280, 268, 251, 239, 229, 208, 198, 171, 159, 144, 137, 130 $cm^{-1}$</td></tr>
<tr><td rowspan="2">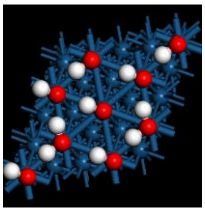</td><td>-</td><td>-</td><td>0.33</td><td>0.67</td><td>-3.782</td><td>0.336</td></tr>
<tr><td colspan="4">$d_{Pt-O(H)}$: 1.998, 2.078 Å<br>$d_{Pt-O(H,H)}$: 2.155 Å<br>$d_{(Pt)O-H}$: 0.981, 0.983 Å<br>$d_{(H)O-H}$: 1.014, 1.015 Å<br>$\angle_{Pt-O-H}$: 104.4°, 106.4°<br>$\angle_{H-O-H}$: 106.4°<br>$\angle_{\text{plane through H-O-H and surface}}$: 13.9°<br>($H_2O$, OH on atop on √3x√3)</td><td colspan="2">3566, 3540, 2964, 2888, 1531, 1124, 1075, 1012, 991, 877, 514, 507, 426, 420, 380, 351, 300, 229, 211, 135, 134 $cm^{-1}$</td></tr>
<tr><td rowspan="2">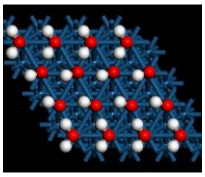</td><td>-</td><td>-</td><td>0.33</td><td>0.67</td><td>-4.049</td><td>0.330</td></tr>
<tr><td colspan="4">$d_{Pt-O(H)}$: 2.045, 2.045, 2.045, 2.046, 2.046, 2.046 Å<br>$d_{Pt-O(H,H)}$: 2.112, 2.112, 2.112 Å<br>$d_{(Pt)O-H}$: 0.986, 0.986, 0.986, 0.987, 0.987, 0.987 Å<br>$d_{(Pt,H)O-H)}$:1.030, 1.031, 1.031, 1.032, 1.032, 1.032 Å<br>$\angle_{Pt-O-H}$: 104.2°, 104.2°, 104.2°, 104.3°, 104.3°, 104.3°<br>$\angle_{H-O-H}$: 110.6°, 110.6°, 110.7°<br>$\angle_{\text{plane through H-O-H and surface}}$: 14.9°, 14.9°, 14.9°<br>($H_2O$, OH on atop on 3x3)</td><td colspan="2">3525, 3523, 3495, 3458, 3248, 3227, 2894, 2870, 2455, 2429, 1547, 1542, 1500, 1174, 1169, 1132, 1102, 1065, 1041, 1036, 1034, 986, 981, 818, 742, 675, 668, 660, 602, 595, 506, 497, 463, 444, 437, 435, 386, 378, 359, 316, 300, 296, 294, 276, 265, 254, 249, 246, 230, 223, 210, 187, 185, 167, 148, 145, 100 $cm^{-1}$</td></tr>
<tr><td rowspan="2">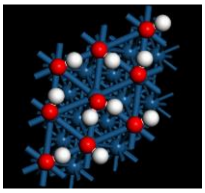</td><td>-</td><td>-</td><td>0.25</td><td>0.75</td><td>-3.927</td><td>0.304</td></tr>
<tr><td colspan="4">$d_{Pt-O(H)}$: 2.008, 2.028, 2.036 Å<br>$d_{Pt-O(H,H)}$: 2.126 Å<br>$d_{(Pt)O-H}$: 1.004, 1.005, 1.016 Å<br>$d_{(H)O-H}$: 1.023, 1.033 Å<br>$\angle_{Pt-O-H}$: 101.9°, 102.9°,<br>$\angle_{H-O-H}$: 97.6°<br>$\angle_{\text{plane through H-O-H and surface}}$: 9.9°<br>($H_2O$, OH on atop on 2x2)</td><td colspan="2">3125, 3086, 2855, 2817, 2612, 1579, 1244, 1194, 1172, 1137, 1107, 1065, 865, 718, 544, 502, 493, 462, 426, 377, 319, 308, 253, 237, 182, 153, 151$cm^{-1}$</td></tr>
</table>

**Table S.2 (cont.)**

| | $\Theta_*$ | $\Theta_O$ | $\Theta_{H2O}$ | $\Theta_{OH}$ | $E_{ads}$, eV | $\Delta_{ads}ZPE$, eV |
|---|---|---|---|---|---|---|
| 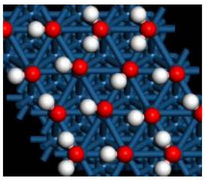 | - | - | 0.22 | 0.77 | -3.800 | 0.291 |
| | $d_{Pt\text{-}O(H)}$: 1.982, 2.005, 2.008, 2.024, 2.024, 2.025, 2.026 Å<br>$d_{Pt\text{-}O(H,H)}$: 2.079, 2.146 Å<br>$d_{(Pt)O\text{-}H}$: 0.987, 0.990, 0.991, 0.996, 0.997, 0.997, 0.997 Å<br>$d_{(H)O\text{-}H}$: 1.021, 1.021, 1.047, 1.051 Å<br>$\angle_{Pt\text{-}O\text{-}H}$: 102.7°, 102.9°, 103.1°, 103.4°, 104.3°, 104.4°, 107.1°<br>$\angle_{H\text{-}O\text{-}H}$: 109.1°, 112.0°<br>$\angle_{\text{plane through H-O-H and surface}}$: 9.7°, 15.3°<br>($H_2O$, OH on atop on 3x3) | | | | 3518, 3462, 3435, 3329, 3298, 3287, 3204, 2863, 2716, 2342, 2211, 1540, 1493, 1327, 1248,<br>1176, 1157, 1144, 1125, 1120, 1116, 1103, 1099, 1093, 957, 838, 714, 697, 632, 564, 543, 531, 488, 477, 473, 470, 461, 450, 450, 435, 429, 375, 333, 316, 314, 303, 291, 289, 279, 276, 261, 245, 217, 213, 201, 187, 179, 159, 144, 26 $cm^{-1}$ | |
| 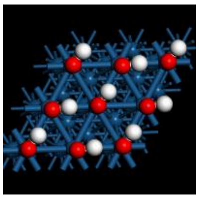 | - | - | - | 1.00 | -3.369 | 0.250 |
| | $d_{Pt\text{-}O(H)}$: 1.975, 1.976, 1.976, 1.976 Å<br>$d_{(Pt)O\text{-}H}$: 1.003, 1.003, 1.003, 1.004 Å<br>$\angle_{Pt\text{-}O\text{-}H}$: 104.4°, 104.4°, 104.4°, 104.4°<br>($H_2O$, OH on atop on 2x2) | | | | 3141, 3137, 3133, 3107, 1209, 1164, 1164, 1154, 747, 744, 717, 543, 512, 512, 511, 493, 312, 304, 302, 243, 224, 223, 166, 165 $cm^{-1}$ | |

**Table S.3:** Geometric characteristics, energetics, and normal modes of surface structures of proposed intermediates in the aerobic oxidation of methane on Pt(111)

| | | | |
|---|---|---|---|
| 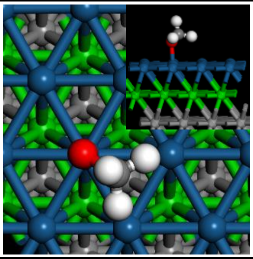 | Methoxy<br>$d_{Pt-O(C)}$: 1.990 Å<br>$d_{(Pt)O-C}$: 1.404 Å<br>$d_{(O)C-H}$: 1.104 Å, 1.112 Å, 1.114 Å<br>$\angle_{Pt-O-C}$: 116.5°<br>$\angle_{O-C-H}$: 106.8°, 112.3°, 112.3° | $E_{ads}$, eV | -2.154 |
| | | $\Delta_{ads}$ZPE, eV | 0.084 |
| | | 2975, 2821, 2796, 1389, 1373, 1362, 1084, 1079, 1004, 471, 259, 235, 131, 81, 47 cm$^{-1}$ | |
| 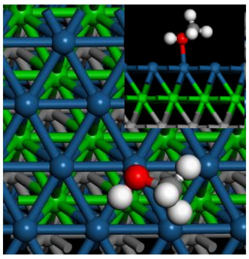 | Methanol<br>$d_{Pt-O}$: 2.290 Å<br>$d_{O-H}$: 0.982 Å<br>$d_{O-C}$: 1.452 Å<br>$d_{C-H}$: 1.095, 1.099, 1.101 Å<br>$\angle_{Pt-O-H}$: 96.1°<br>$\angle_{Pt-O-C}$: 117.3°<br>$\angle_{H-O-C}$: 109.3°<br>$\angle_{O-C-H}$: 106.2°, 108.8°, 110.9° | $E_{ads}$, eV | -1.702 |
| | | $\Delta_{ads}$ZPE, eV | 0.185 |
| | | 3583, 3087, 3035, 2976, 1440, 1430, 1401, 1297, 1123, 1058, 947, 576, 272, 215, 175, 140, 115, 58 cm$^{-1}$ | |
| 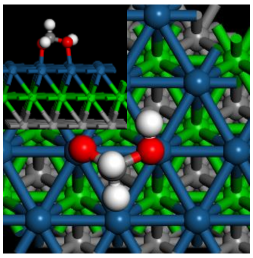 | Hydroxy-methoxy<br>$d_{Pt-O(C)}$: 2.048 Å<br>$d_{Pt-O(C,H)}$: 2.238 Å<br>$d_{(Pt)O-H}$: 0.982 Å<br>$d_{(Pt,H)O-C}$: 1.499 Å<br>$d_{(Pt)O-C}$: 1.351 Å<br>$d_{C-H}$: 1.105 Å, 1.114 Å<br>$\angle_{Pt-O(H)-C}$: 111.7°<br>$\angle_{Pt-O-C}$: 115.9°<br>$\angle_{Pt-O-H}$: 101.1°<br>$\angle_{O-C-O}$: 113.9°<br>$\angle_{O-C-H}$: 101.6°, 105.7°<br>$\angle_{O(H)-C-H}$: 108.8°, 115.1° | $E_{ads}$, eV | -4.388 |
| | | $\Delta_{ads}$ZPE, eV | 0.197 |
| | | 3602, 2958, 2790, 1401, 1329, 1248, 1172, 1106, 987, 742, 603, 487, 408, 303, 248, 138, 104, 84 cm$^{-1}$ | |
| 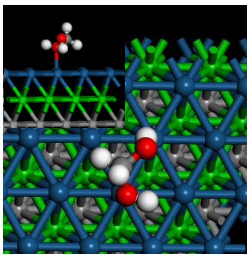 | Methanediol<br>$d_{Pt-O(C)}$: 2.264 Å<br>$d_{(Pt,H)-O-C}$: 1.475 Å<br>$d_{(H)-O-C}$: 1.379 Å<br>$d_{(Pt)O-H}$: 0.981 Å<br>$d_{O-H}$: 0.996 Å<br>$d_{C-H}$: 1.099 Å, 1.104 Å<br>$\angle_{Pt-O(H)-C}$:119.3°<br>$\angle_{Pt-O-H}$: 104.4°<br>$\angle_{O-C-O}$: 112.9°<br>$\angle_{O-C-H}$: 103.3°, 106.6°<br>$\angle_{C-O-H}$: 108.8° | $E_{ads}$, eV | -3.967 |
| | | $\Delta_{ads}$ZPE, eV | 0.250 |
| | | 3627, 3268, 3034, 2937, 1441, 1373, 1359, 1269, 1173, 1068, 986, 835, 579, 524, 439, 265, 187, 149, 124, 92, 26 cm$^{-1}$ | |

**Table S.4:** Geometric characteristics, energetics, and normal modes of surface structures of proposed intermediates in the aerobic oxidation of methane on Pt(111) covered with O, OH and $H_2O$

| | | | | |
|---|---|---|---|---|
| Initial structure<br>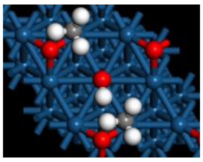<br>$\Theta_{CH3}$ = 0.25<br>$\Theta_{O}$ = 0.25<br>$\Theta_{OH}$ = 0.25 | Final structure<br>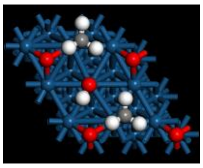<br>$\Theta_{CH3}$ = 0.25<br>$\Theta_{O}$ = 0.25<br>$\Theta_{OH}$ = 0.25 | $d_{Pt\text{-}O}$: 2.043, 2.052, 2.058 Å<br>$d_{Pt\text{-}O(H)}$: 2.007 Å<br>$d_{O\text{-}H}$: 0.978 Å<br>$d_{Pt\text{-}C(H)}$: 2.005 Å<br>$d_{C\text{-}H}$: 1.093, 1.093, 1.094 Å<br>$\angle_{Pt\text{-}O\text{-}H}$: 105.9°<br>$\angle_{Pt\text{-}C\text{-}H}$: 102.7°, 108.3°, 108.4° | $E_{ads}$, eV<br>$\Delta_{ads}$ZPE, eV<br>3669, 3126, 3112, 2993, 1404, 1368, 1200, 953, 845, 825, 505, 499, 471, 407, 365, 268, 226, 193, 168, 141, 102 $cm^{-1}$ | -1.551<br>0.132 |
| Initial structure<br>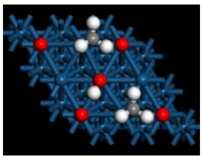<br>$\Theta_{CH3}$ = 0.25<br>$\Theta_{O}$ = 0.25<br>$\Theta_{OH}$ = 0.25 | Final structure<br>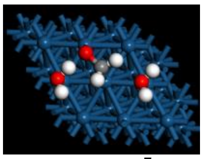<br>$\Theta_{OCH2}$ = 0.50[7]<br>$\Theta_{H2O}$ = 0.25 | $d_{Pt\text{-}O(C)}$: 2.175 Å<br>$d_{Pt\text{-}O(H)}$: 2.221 Å<br>$d_{O\text{-}H}$: 0.975, 1.044 Å<br>$d_{O\text{-}C}$: 1.379 Å<br>$d_{C\text{-}H}$: 1.101, 1.106 Å<br>$\angle_{Pt\text{-}O\text{-}C}$: 103.8°<br>$\angle_{O\text{-}C\text{-}H}$: 112.7°, 113.3°<br>$\angle_{H\text{-}O\text{-}H}$: 111.9°<br>$\angle_{\text{plane through H-O-H and surface}}$: 22.9° | $E_{ads}$, eV<br>$\Delta_{ads}$ZPE, eV<br>3711, 2979, 2902, 2377, 1559, 1399, 1184, 1130, 1106, 956, 725, 679, 546, 511, 356, 326, 276, 251, 202, 166, 139 $cm^{-1}$ | -3.100<br>0.171 |
| Initial structure<br>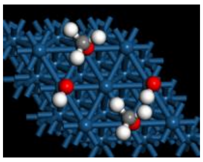<br>$\Theta_{OCH3}$ = 0.25<br>$\Theta_{OH}$ = 0.25 | Final structure<br>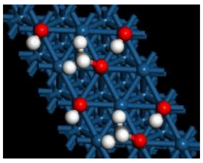<br>$\Theta_{OCH3}$ = 0.25<br>$\Theta_{OH}$ = 0.25 | $d_{Pt\text{-}O(H)}$: 1.980 Å<br>$d_{Pt\text{-}O(C)}$: 2.028 Å<br>$d_{O\text{-}H}$: 1.054 Å<br>$d_{C\text{-}H}$: 1.104, 1.111, 1.118 Å<br>$\angle_{Pt\text{-}O\text{-}H}$: 103.1°<br>$\angle_{Pt\text{-}O\text{-}C}$: 115.1°<br>$\angle_{Pt\text{-}O\text{-}C}$: 107.4° 111.5°, 112.7° | $E_{ads}$, eV<br>$\Delta_{ads}$ZPE, eV<br>3140, 2976, 2846, 2734, 1393, 1373, 1344, 1172, 1089, 1078, 997, 722, 539, 445, 325, 283, 243, 194, 135, 115, 96 $cm^{-1}$ | -2.047<br>0.156 |
| Initial structure<br>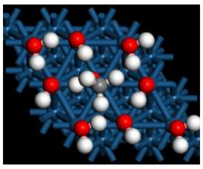<br>$\Theta_{OCH3}$ = 0.25<br>$\Theta_{H2O}$ = 0.50<br>$\Theta_{OH}$ = 0.25 | Final structure<br>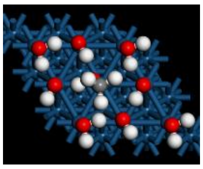<br>$\Theta_{HOCH3}$ = 0.25<br>$\Theta_{H2O}$ = 0.25<br>$\Theta_{OH}$ = 0.50 | $d_{Pt\text{-}O(C)}$: 2.838 Å<br>$d_{(Pt)O\text{-}C}$: 1.430 Å<br>$d_{O\text{-}H}$: 1.022 Å<br>$d_{C\text{-}H}$: 1.097, 1.100, 1.103 Å<br>$\angle_{Pt\text{-}O\text{-}C}$: 146.5°<br>$\angle_{Pt\text{-}O\text{-}H}$: 89.0°<br>$\angle_{O\text{-}C\text{-}H}$: 108.0°, 109.1°, 110.8°<br>$d_{Pt\text{-}O(H)}$: 2.061, 2.064 Å<br>$d_{Pt\text{-}O(H,H)}$: 2.139 Å<br>$d_{(Pt)O\text{-}H}$: 1.006, 1.020 Å<br>$d_{(Pt,H)O\text{-}H}$: 1.032, 1.036 Å<br>$\angle_{Pt\text{-}O\text{-}H}$: 101.8°, 104.9°<br>$\angle_{H\text{-}O\text{-}H}$: 96.7°<br>$\angle_{\text{plane through H-O-H and surface}}$: 6.9° | $E_{ads}$, eV<br>$\Delta_{ads}$ZPE, eV<br>3076, 3058, 3008, 2949, 2827, 2698, 2676, 2499, 1610, 1474, 1445, 1440, 1424, 1256, 1235, 1181, 1157, 1136, 1126, 1102, 1023, 931, 838, 693, 487, 459, 438, 384, 341, 317, 277, 231, 207, 169, 148, 110, 90, 78, 68 $cm^{-1}$ | -3.761<br>0.411 |

[7] Di-σ surface species

**Table S.4 (cont.):**

| Initial structure | Final structure | Geometry | Energetics and frequencies |
|---|---|---|---|
| 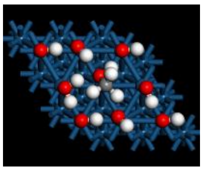<br>$\Theta_{HOCH3}$ = 0.25<br>$\Theta_{H2O}$ = 0.25<br>$\Theta_{OH}$ = 0.50 | 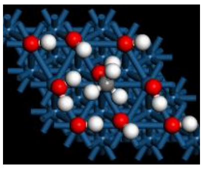<br>$\Theta_{HOCH3}$ = 0.25<br>$\Theta_{H2O}$ = 0.25<br>$\Theta_{OH}$ = 0.50 | $d_{Pt-O(C)}$: 3.185 Å<br>$d_{(Pt)O-C}$: 1.430 Å<br>$d_{O-H}$: 1.025 Å<br>$d_{C-H}$: 1.098, 1.099, 1.102 Å<br>$\angle_{Pt-O-C}$: 137.7°<br>$\angle_{Pt-O-H}$: 73.3°<br>$\angle_{O-C-H}$: 108.0°, 110.0°, 110.9°<br>$d_{Pt-O(H)}$: 2.041, 2.056 Å<br>$d_{Pt-O(H,H)}$: 2.148 Å<br>$d_{(Pt)O-H}$: 0.987, 1.030 Å<br>$d_{(Pt,H)O-H}$: 1.000, 1.046 Å<br>$\angle_{Pt-O-H}$: 99.9°, 110.4°<br>$\angle_{H-O-H}$: 98.8°<br>$\angle_{\text{plane through H-O-H and surface}}$: 14.4° | $E_{ads}$, eV: -3.804<br>$\Delta_{ads}$ZPE, eV: 0.406<br>3437, 3243, 3052, 3023, 2957, 2734, 2556, 2366, 1620, 1507, 1453, 1440,<br>1415, 1276, 1192, 1143, 1135, 1073, 1036, 1004, 973, 924, 776, 528, 474, 454, 435, 417, 337, 295, 266, 217, 168, 149, 134, 109, 103, 90, 74 $cm^{-1}$ |
| 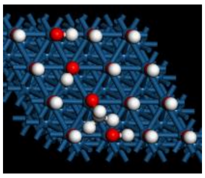<br>$\Theta_{OCH3}$ = 0.11<br>$\Theta_{OH}$ = 0.88 | 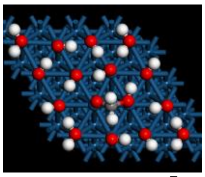<br>$\Theta_{OCH2OH}$ = 0.22[7]<br>$\Theta_{H2O}$ = 0.11<br>$\Theta_{OH}$ = 0.66 | $d_{Pt-O(C)}$: 2.039 Å<br>$d_{Pt-O(C,H)}$: 2.180 Å<br>$d_{(Pt)O-C}$: 1.371 Å<br>$d_{(Pt,H)O-C}$: 1.471 Å<br>$d_{O-H}$: 1.013 Å<br>$d_{C-H}$: 1.098, 1.104 Å<br>$\angle_{Pt-O-C}$: 124.8°<br>$\angle_{Pt-O(H)-C}$: 116.1°<br>$\angle_{Pt-O-H}$: 102.2°<br>$\angle_{O-C-O}$: 115.7°<br>$d_{Pt-O(H)}$: 1.979, 1.981, 1.987, 2.020, 2.045, 2.055 Å<br>$d_{Pt-O(H,H)}$: 2.097 Å<br>$d_{(Pt)O-H}$: 0.984, 0.994, 1.000, 1.003, 1.008, 1.015 Å<br>$d_{(Pt,H)O-H}$: 1.001, 1.050 Å<br>$\angle_{Pt-O-H}$: 100.0°, 100.6°,101.5°, 103.1°, 104.1°, 105.5°<br>$\angle_{H-O-H}$: 111.4°<br>$\angle_{\text{plane through H-O-H and surface}}$: 19.5° | $E_{ads}$, eV: -4.740<br>$\Delta_{ads}$ZPE, eV: 0.320<br>3625, 3374, 3268, 3239, 3205, 3021, 2947, 2912, 2894, 2817, 2222, 1540, 1406, 1352, 1308, 1260, 1205, 1197, 1188, 1175, 1165, 1082, 1062, 1052, 1048, 1026, 959, 850, 831, 800, 774, 712, 642, 607, 561, 515, 507, 504, 477, 445, 434, 425, 410, 379, 375, 366, 334, 328, 312, 294, 276, 269, 263, 258, 254, 227, 202, 193, 184, 172, 152, 138, 129 $cm^{-1}$ |
| 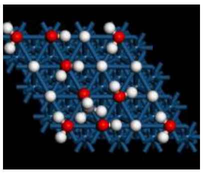<br>$\Theta_{OCH3}$ = 0.11<br>$\Theta_{H2O}$ = 0.11<br>$\Theta_{OH}$ = 0.77 | 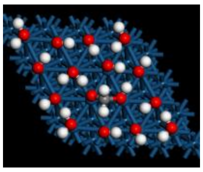<br>$\Theta_{OCH2OH}$ = 0.22[7]<br>$\Theta_{H2O}$ = 0.22<br>$\Theta_{OH}$ = 0.55 | $d_{Pt-O(C)}$: 2.078 Å<br>$d_{Pt-O(C,H)}$: 2.184 Å<br>$d_{(Pt)O-C}$: 1.382 Å<br>$d_{(Pt,H)O-C}$: 1.474 Å<br>$d_{O-H}$: 1.013 Å<br>$d_{C-H}$: 1.094, 1.101 Å<br>$\angle_{Pt-O-C}$: 126.5°<br>$\angle_{Pt-O(H)-C}$: 119.4°<br>$\angle_{Pt-O-H}$: 102.8°<br>$\angle_{O-C-O}$: 115.3°<br>$d_{Pt-O(H)}$: 1.992, 2.036, 2.043, 2.055, 2.066 Å<br>$d_{Pt-O(H,H)}$: 2.105, 2.126 Å<br>$d_{(Pt)O-H}$: 0.993, 0.997, 1.010, 1.011, 1.016 Å<br>$d_{(Pt,H)O-H}$: 1.003, 1.003, 1.050, 1.056 Å<br>$\angle_{Pt-O-H}$: 100.1°, 101.1°, 101.8°, 102.1°, 103.8°<br>$\angle_{H-O-H}$: 101.8°, 106.9°<br>$\angle_{\text{plane through H-O-H and surface}}$: 12.8°, 15.7° | $E_{ads}$, eV: -4.866<br>$\Delta_{ads}$ZPE, eV: 0.355<br>3497, 3411, 3267, 3181, 3104, 3087, 2951, 2935, 2587, 2771, 2363, 2113,<br>1624, 1566, 1412, 1379, 1323, 1270, 1260, 1239, 1227, 1199, 1161, 1111,<br>1096, 1084, 1072, 1058, 1036, 980, 966, 927, 865,<br>803, 760, 698, 626, 576, 546, 504, 464, 449, 440, 425, 408, 405, 378, 357,<br>354, 332, 312, 308, 306, 292, 283, 273, 264, 244, 223, 203, 191, 183, 180,<br>143, 130, 117 $cm^{-1}$ |

**Table S.4 (cont.)**

| | | | |
|---|---|---|---|
| Initial structure<br>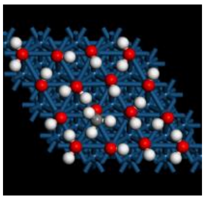<br>$\Theta_{OCH3}$ = 0.11<br>$\Theta_{H2O}$ = 0.33<br>$\Theta_{OH}$ = 0.55 | Final structure<br>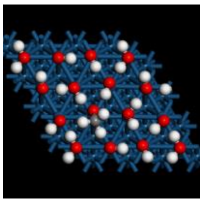<br>$\Theta_{OCH3}$ = 0.11<br>$\Theta_{H2O}$ = 0.33<br>$\Theta_{OH}$ = 0.55 | $d_{Pt-O(C)}$: 2.133 Å<br>$d_{(Pt)O-C}$: 1.431 Å<br>$d_{C-H}$: 1.101, 1.01, 1.103 Å<br>$\angle_{Pt-O-C}$: 132.3°<br>$\angle_{O-C-H}$: 108.0°, 110.1°, 110.4°<br>$d_{Pt-O(H)}$: 1.973, 2.040, 2.056 Å<br>2.075, 2.077 Å<br>$d_{Pt-O(H,H)}$: 2.080, 2.106, 1.141 Å<br>$d_{(Pt)O-H}$: 0.985, 1.000, 1.001,<br>1.003, 1.005 Å<br>$d_{(Pt,H)O-H}$: 0.983, 1.024, 1.033,<br>1.038, 1.047, 1.055 Å<br>$\angle_{Pt-O-H}$: 100.5°, 101.0°, 101.3°<br>102.6°, 103.6°<br>$\angle_{H-O-H}$: 111.4°, 111.7°, 112.4°<br>$\angle_{\text{plane through H-O-H and surface}}$: 17.0°, 17.2°, 25.9° | $E_{ads}$, eV: -4.198<br>$\Delta_{ads}$ZPE, eV: 0.586<br>3596, 3449, 3274, 3219, 3111, 3065, 3034, 2989, 2913, 2781, 2645, 2418,<br>2258, 2240, 1562, 1527, 1487, 1452, 1432, 1386, 1296, 1228, 1201, 1199, 1186, 1159, 1152, 1139, 1132, 1124, 1078, 1047, 1005, 992, 948, 925, 867, 793, 745, 659, 576, 557, 514, 485, 453, 447, 431, 416, 408, 401, 374, 362, 358, 348, 340, 310, 306, 297, 276, 269, 256, 246, 235, 231, 224, 214, 187, 178, 172, 147, 128, 119 $cm^{-1}$ |
| Initial structure<br>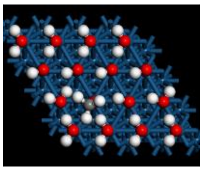<br>$\Theta_{OCH3}$ = 0.11<br>$\Theta_{H2O}$ = 0.44<br>$\Theta_{OH}$ = 0.44 | Final structure<br>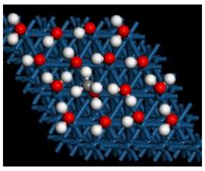<br>$\Theta_{HOCH3}$ = 0.11<br>$\Theta_{H2O}$ = 0.33<br>$\Theta_{OH}$ = 0.55 | $d_{Pt-O(C,H)}$: 2.969 Å<br>$d_{(Pt)O-C}$: 1.432 Å<br>$d_{O-H}$: 1.017 Å<br>$d_{C-H}$: 1.097, 1.100, 1.102 Å<br>$d_{Pt-O(H)}$: 2.050, 2.059, 2.068,<br>2.071, 2.094 Å<br>$d_{Pt-O(H,H)}$:2.089, 2.137, 2.163 Å<br>$d_{(Pt)O-H}$: 0.985, 0.990, 0.990,<br>0.998, 1.011 Å<br>$d_{(Pt,H)O-H}$: 0.990, 1.018, 1.028,<br>1.055, 1.058, 1.075 Å<br>$\angle_{Pt-O-H}$: 101.6°, 102.0°,102.2°,<br>102.3°, 104.0°<br>$\angle_{H-O-H}$: 97.6°, 107.5°, 111.3°<br>$\angle_{\text{plane through H-O-H and surface}}$: 9.6°, 14.5°, 26.9° | $E_{ads}$, eV: -4.407<br>$\Delta_{ads}$ZPE, eV: 0.381<br>3696, 3508, 3417, 3396, 3295, 3132, 3087, 3064, 3037, 2966, 2861, 2653, 2593, 2573, 2312, 1579, 1533, 1515, 1480, 1452, 1445, 1422, 1224, 1223, 1211, 1143, 1142, 1140, 1114, 1108, 1085, 1074, 1054, 1046, 1019, 1005, 933, 899, 788, 775, 756, 704, 658, 528, 455, 452, 440, 430, 419, 411, 402, 396, 354, 352, 335. 330, 315, 301, 289, 277, 274, 262, 240, 215, 214, 203, 200, 186, 135, 132, 126, 113, 94, 76, 29 $cm^{-1}$ |
| Initial structure<br>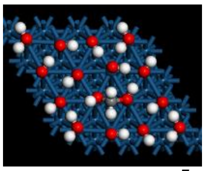<br>$\Theta_{HOCH2OH}$ = 0.22[7]<br>$\Theta_{H2O}$ = 0.11<br>$\Theta_{OH}$ = 0.66 | Final structure<br>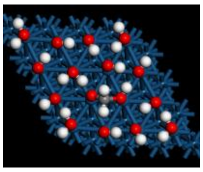<br>$\Theta_{OCH2OH}$ = 0.22[7]<br>$\Theta_{H2O}$ = 0.22<br>$\Theta_{OH}$ = 0.55 | $d_{Pt-O(C)}$: 2.078 Å<br>$d_{Pt-O(C,H)}$: 2.184 Å<br>$d_{(Pt)O-C}$: 1.382 Å<br>$d_{(Pt,H)O-C}$: 1.474 Å<br>$d_{O-H}$: 1.013 Å<br>$d_{C-H}$: 1.094, 1.101 Å<br>$\angle_{Pt-O-C}$: 126.5°<br>$\angle_{Pt-O(H)-C}$: 119.4°<br>$\angle_{Pt-O-H}$: 102.8°<br>$\angle_{O-C-O}$: 115.3°<br>$d_{Pt-O(H)}$: 1.992, 2.036, 2.043,<br>2.055, 2.066 Å<br>$d_{Pt-O(H,H)}$: 2.105, 2.126 Å<br>$d_{(Pt)O-H}$: 0.993, 0.997, 1.010,<br>1.011, 1.016 Å<br>$d_{(Pt,H)O-H}$: 1.003, 1.003, 1.050,<br>1.056 Å<br>$\angle_{Pt-O-H}$: 100.1°, 101.1°,<br>101.8°, 102.1°, 103.8°<br>$\angle_{H-O-H}$: 101.8°, 106.9°<br>$\angle_{\text{plane through H-O-H and surface}}$: 12.8°, 15.7° | $E_{ads}$, eV: -4.866<br>$\Delta_{ads}$ZPE, eV: 0.355[8] |

[8] Not determined as structure is identical to structure starting from $\Theta_{OCH3}$ = 0.11, $\Theta_{H2O}$ = 0.11 and $\Theta_{OH}$ = 0.77

**References**


[S1] C.A. Beach, C. Krumm, C.S. Spanjers, S. Maduskar, A.J. Jones, P.J. Dauenhauer: Quantitative carbon detector for enhanced detection of molecules in foods, pharmaceuticals, cosmetics, flavors, and fuels. *Analyst* **2016**, *141*, 1627-1632.

[S2] G. Kresse, J. Hafner: Ab initio molecular dynamics for liquid metals. *Physical Review B Condensed Matter* **1993**, *47*, 558-561.

[S3] G. Kresse, J. Furthmüller: Efficient iterative schemes for ab initio total-energy calculations using a plane-wave basis set. *Physical Review B* **1996**, *54*, 11169–11186.

[S4] G. Kresse, D. Joubert: From ultrasoft pseudopotentials to the projector augmented-wave method. *Physical Review B* **1999**, *59*, 1758–1775.

[S5] J.P. Perdew, K. Burke, M. Ernzerhof: Generalized gradient approximation made simple. *Physical Review Letters* **1996**, *77* (1996), 3865-3868.

[S6] S. Grimme, J. Antony, S. Ehrlich, S. Krieg: A consistent and accurate *ab initio* parametrization of density functional dispersion correction (DFT-D) for the 94 elements H-Pu. *Journal of Chemical Physics* **2010**, *132*, 154104.

[S7] S. Grimme, S. Ehrlich, L. Goerigk: Effect of the damping function in dispersion corrected density functional theory. *Journal of Computational Chemistry* **2011**, *32*, 1456-1465.

[S8] M. Methfessel, A.T. Paxton: High precision sampling for Brillouin zone integration in metals. *Physical Review B.* **1989**, *40*, 3616-3621.

[S9] H.J. Monkhorst, J.D. Pack: Special points for Brillouin-zone integration. *Physical Review B* **1976**, *13*, 5188-5192.

[S10] W.M. Haynes, D.R. Lide, Eds.: *Handbook of Chemistry and Physics*. 91st ed., CRC Press, Boca Raton, **2010**.

[S11] NIST Chemistry Webbook (https://webbook.nist.gov/cgi/cbook.cgi)

[S12] D.R. Kent IV, S.L. Widicus, G.A. Blake, W.A. Goddard III: A theoretical study of the conversion of gas phase methanediol to formaldehyde. *Journal of Chemical Physics* **2003**, *119*, 5117-5120.

[S13] J. Rossmeisl, A. Logadottir, J.K. Nørskov: Electrolysis of water on (oxidized) metal surfaces. *Chemical Physics* **2005**, *319*, 178–184.

[S14] E.M. Karp, C.T. Campbell, F. Studt, F. Abild-Pedersen, J.K. Nørskov: Energetics of oxygen adatoms, hydroxyl species and water dissociation on Pt(111). *The Journal of Physical Chemistry C* **2012**, *116(49)*, 25772-25775.
